# Cost-effectiveness of World Health Organization 2010 Guidelines for Prevention of Mother-to-Child HIV Transmission in Zimbabwe

**DOI:** 10.1093/cid/cis858

**Published:** 2012-11-30

**Authors:** Andrea L. Ciaranello, Freddy Perez, Barbara Engelsmann, Rochelle P. Walensky, Angela Mushavi, Asinath Rusibamayila, Jo Keatinge, Ji-Eun Park, Matthews Maruva, Rodrigo Cerda, Robin Wood, Francois Dabis, Kenneth A. Freedberg

**Affiliations:** 1 Medical Practice Evaluation Center, Divisions of Infectious Disease; 2 Division of General Medicine; 3 Department of Medicine, Massachusetts General Hospital; 4 Division of Infectious Disease, Brigham and Women's Hospital; 5 Center for AIDS Research, Harvard Medical School, Boston, Massachusetts; 6 Universite Bordeaux, Institut de Santé Publique, d'Epidémiologie et de Développement, and Centre INSERM U897-Epidemiologie-Biostatistique, Bordeaux, France; 7 HIV/AIDS Unit, Pan American Health Organization, Washington, DC; 8 Organisation for Public Health Interventions and Development; 9 Ministry of Health and Child Welfare; 10 United States Agency for International Development, Harare, Zimbabwe; 11 Desmond Tutu HIV Centre, Institute of Infectious Disease and Molecular Medicine, University of Cape Town, South Africa

**Keywords:** HIV, mother-to-child transmission, PMTCT, pediatric HIV, cost-effectiveness

## Abstract

***Background.*** In 2010, the World Health Organization (WHO) released revised guidelines for prevention of mother-to-child human immunodeficiency virus (HIV) transmission (PMTCT). We projected clinical impacts, costs, and cost-effectiveness of WHO-recommended PMTCT strategies in Zimbabwe.

***Methods.*** We used Zimbabwean data in a validated computer model to simulate a cohort of pregnant, HIV-infected women (mean age, 24 years; mean CD4 count, 451 cells/µL; subsequent 18 months of breastfeeding). We simulated guideline-concordant care for 4 PMTCT regimens: single-dose nevirapine (sdNVP); WHO-recommended Option A, WHO-recommended Option B, and Option B+ (lifelong maternal 3-drug antiretroviral therapy regardless of CD4). Outcomes included maternal and infant life expectancy (LE) and lifetime healthcare costs (2008 US dollars [USD]). Incremental cost-effectiveness ratios (ICERs, in USD per year of life saved [YLS]) were calculated from combined (maternal + infant) discounted costs and LE.

***Results.*** Replacing sdNVP with Option A increased combined maternal and infant LE from 36.97 to 37.89 years and would reduce lifetime costs from $5760 to $5710 per mother–infant pair. Compared with Option A, Option B further improved LE (38.32 years), and saved money within 4 years after delivery ($5630 per mother–infant pair). Option B+ (LE, 39.04 years; lifetime cost, $6620 per mother–infant pair) improved maternal and infant health, with an ICER of $1370 per YLS compared with Option B.

***Conclusions.*** Replacing sdNVP with Option A or Option B will improve maternal and infant outcomes and save money; Option B increases health benefits and decreases costs compared with Option A. Option B+ further improves maternal outcomes, with an ICER (compared with Option B) similar to many current HIV-related healthcare interventions.

## Abstract (precis)

We projected outcomes for mothers and infants following World Health Organization–recommended regimens to prevent mother-to-child human immunodeficiency virus (HIV) transmission. Compared with Option A, Option B improves life expectancy and saves money; compared with Option B, lifelong maternal therapy is of comparable value to common HIV-related interventions.

**(See the Editorial Commentary by Sawe and Lockman on pages 447–9.)**

Effective medications for the prevention of mother-to-child human immunodeficiency virus (HIV) transmission (PMTCT) can reduce perinatal HIV transmission to <2% in the absence of breastfeeding and to <5% by 6 months of age among breastfeeding infants [1–3]. As a result, the World Health Organization (WHO) has called for the “virtual elimination” of pediatric HIV [1–3]. Access to antiretroviral medications (ARVs) for PMTCT remains limited, however; only 59% of HIV-infected pregnant women received ARVs for PMTCT in 2010 [4]. As a result, nearly 400 000 new infant HIV infections occur annually, and HIV-infected women experience high postpartum morbidity and mortality [4–6].

In 2010, WHO issued revised guidelines for PMTCT [1]. The guidelines included a renewed emphasis on identification of pregnant, HIV-infected women with CD4 count ≤350 cells/µL or WHO stage 3–4 disease, who require lifelong 3-drug antiretroviral therapy (ART) for treatment of their own HIV infections and for PMTCT. For women with less-advanced disease, WHO recommends a country- or program-level choice between Option A (maternal zidovudine in pregnancy; infant nevirapine [NVP] throughout breastfeeding), and Option B (maternal 3-drug ARV regimens throughout pregnancy and breastfeeding, with interruption after weaning). Select programs are considering Option B+, in which maternal 3-drug regimens are initiated in pregnancy (regardless of maternal CD4) and continued throughout life, including throughout breastfeeding and subsequent pregnancies [3, 7].

HIV prevalence in antenatal care (ANC) is estimated at 16% in Zimbabwe, leading to approximately 61 000 births per year to HIV-infected women [8, 9]. Through 2009, the Zimbabwe National PMTCT Program provided single-dose NVP (sdNVP) to all HIV-infected women, with ART for women identified clinically as ART eligible [8]. Like most countries in sub–Saharan Africa, Zimbabwe initially implemented the revised WHO guidelines with Option A (with antenatal coverage of 46% in 2010) and will soon be examining the feasibility of Options B and B+ [4]. We used validated computer models of HIV disease and PMTCT [10–12] to project the clinical outcomes and cost effectiveness of implementing WHO-recommended PMTCT regimens in Zimbabwe.

## 

## METHODS

### Analytic Overview

**Figure 1.**
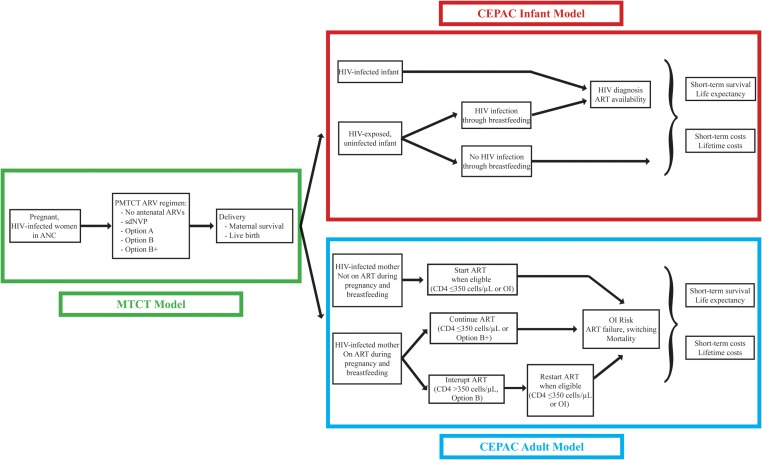
Model structure. Three linked models were used for this analysis, as described in the Methods, as well as in the Supplementary Appendix and previous work [10, 14, 15]. The mother-to-child human immunodeficiency virus transmission model includes events during pregnancy and delivery (left panel; Supplementary Figure 1). The Cost-effectiveness of Preventing AIDS Complications (CEPAC) adult model includes events occurring among mothers after delivery (bottom right panel; Supplementary Figure 2*A*), and the CEPAC infant model includes events for infants after birth (top right panel; Supplementary Figure 2*B*). Linkages between the models allow a combined analysis in which each woman–infant pair is simulated together from the time of first presentation at antenatal care through pregnancy and delivery, and then each woman and infant are simulated separately throughout their lifetimes. Abbreviations: ANC, antenatal care; ART, 2-drug antiretroviral therapy; ARVs, antiretroviral medications; HIV, human immunodeficiency virus; OI, opportunistic infection; PMTCT, prevention of mother-to-child HIV transmission; sdNVP, single-dose nevirapine.

We used 3 validated, linked computer models for this analysis (Figure 1): (1) a model of a single pregnancy and delivery (the mother-to-child HIV transmission [MTCT] model [10]); (2) the Cost-effectiveness of Preventing AIDS Complications (CEPAC) model of HIV infection and mortality among breastfed infants (the CEPAC infant model [13, 14]); and (3) the CEPAC-International model of HIV disease progression among postpartum women (the CEPAC adult model [11, 12, 15]). Clinical outcomes of the linked models included infant HIV infection risk at weaning, maternal life expectancy (LE) from delivery, and infant LE from birth. Economic outcomes, from the healthcare system perspective, included ANC costs (through delivery), maternal HIV-related healthcare costs, and infant healthcare costs.

Incremental cost-effectiveness ratios (ICERs), in US dollars per year of life saved (YLS), were calculated from combined projected lifetime healthcare costs (antenatal + maternal + infant) and combined projected life expectancy (maternal + infant) [16], discounted at 3% per year. We used 2 criteria to interpret cost-effectiveness. First, following WHO guidance, an intervention was considered cost-effective if its ICER compared with the next least-expensive alternative was <3 times the 2008 Zimbabwe per capita gross domestic product, or 3 × $400 = $1200 per YLS [17, 18]. Second, we compared results with the recently reported range of ICERs for ART-related interventions in developing countries ($550–$5200 per YLS) [19]. This work was approved by the Partners Healthcare Institutional Review Board, Boston, Massachusetts.

### 

### Modeled Population, PMTCT Regimens, and Uptake of PMTCT Services

The linked models were used to simulate a cohort of pregnant, HIV-infected women in Zimbabwe and their infants. We examined 5 PMTCT regimens: (1) no antenatal ARVs (comparator), (2) sdNVP, (3) WHO Option A, (4) WHO Option B, and (5) Option B+ (Supplementary Table 1). Women were modeled to present to care at 24–28 weeks’ gestation and to breastfeed for 18 months, based on Zimbabwean data [20, 21], with ARV prophylaxis (Options A, B, and B+ ) continued throughout breastfeeding.

To demonstrate the impact of guideline-concordant care, all women in the base-case analyses were assumed to be identified as HIV-infected at their first ANC visit. With no ARVs, women received no antiretroviral medications during pregnancy. With sdNVP, women initiated ART in pregnancy if clinical assessment indicated WHO stage 3–4 disease; CD4 testing was not included, reflecting its limited availability in the sdNVP-based National PMTCT Program in 2009. With Options A and B, women received ART during pregnancy if eligible by either CD4 or clinical criteria, and with Option B+, all women received lifelong ART. With all modeled regimens, women who linked to postnatal HIV care were assumed to undergo clinical and CD4 assessment at 6 weeks postpartum and to initiate ART if eligible, regardless of antenatal regimen received. In the base case, we assumed 100% adherence to PMTCT regimens (initiated at 30 weeks’ gestational age), 100% linkage to postnatal care for mothers and infants, and 100% retention in care and ART availability for women and infants meeting WHO ART initiation criteria [22, 23]. In sensitivity analyses, to reflect real-world programs, we examined reduced access to antenatal and postnatal care.

### 

### Model Structure

The 3 simulation models are described in detail in the Supplementary Appendix and in previous publications [10, 14, 15]. The models were linked so that each mother–infant pair was simulated together from the time of first presentation at ANC through delivery (the MTCT model), and then each woman and infant were simulated separately over their lifetimes after delivery (the CEPAC adult and infant models), as in Figure 1 and Supplementary Figures 1 and 2.

### 

### Model Input Parameters

#### Maternal Characteristics, Disease Progression, and ART

Based on Zimbabwean data, mean age at first ANC visit was 24 years [21]; mean CD4 count was 451 cells/µL (36% of women with CD4 count ≤350 cells/µL) [24]. Because detailed data to inform monthly risks of opportunistic infections (OIs) and HIV-related death in the absence of ART were not available from Zimbabwe, we derived these data from a cohort in South Africa (Supplementary Table 2) [25]. Details of ART initiation and switching, as well as CD4 and HIV RNA changes on ART, are provided in the Supplementary Appendix.

#### 

#### MTCT Risks, Infant Mortality Rates, and Infant Life Expectancy Estimates

Risks of MTCT during pregnancy and breastfeeding were calculated from PMTCT studies among breastfeeding populations in Africa, leading to estimates similar to those derived by the Joint United Nations Programme on HIV/AIDS (Supplementary Appendix) [10, 26]. Data and assumptions to inform infant mortality rates and LE values are shown in Table 1 and detailed in the Supplementary Appendix.

**Table 1.** Selected Model Input Parameters

Variable	Value	Data Sources			
Clinical Model Input Parameters					
Baseline Maternal Cohort Characteristics					
Age, mean, y (SD)	24 (5)	MOHCW [21]			
Mortality during pregnancy	0.7%	MOHCW [8]			
Proportion ART eligible^a^	36%	ZVITAMBO trial [24]			
CD4 count, cells/µL (SD)					
Total cohort	451 (50)	ZVITAMBO trial [24]			
ART-eligible women	275 (50)	ZVITAMBO trial [24]			
Non-ART-eligible women	550 (50)	ZVITAMBO trial [24]			
Uptake of PMTCT services and postnatal care					
PMTCT uptake^b^	100% (sensitivity analyses: 56%, 80%, 95%)	WHO [1]			
Sensitivity of clinical assessment of ART eligibility	36%	MTCT-Plus Cohort [47]			
Probability of linking to pediatric HIV diagnosis, care, and ART	100% (sensitivity analysis: 36%)	WHO/UNICEF [48]			
Probability of linking to postnatal maternal HIV-related care	100% (sensitivity analyses: 87% if ANC received, 43% if no ANC received)	After ANC: Mean of published values [49–54] No ANC: assumption			
Loss to follow-up from postnatal maternal care	0% per year (sensitivity analyses: 16% [year 1]; 6% per year [years ≥2])	[30–32]			
	Base Case Value (range for sensitivity analysis)				
Maternal HIV Status					
Mother-to-Child Transmission Risks	PMTCT Regimen Received				
Intrauterine/intrapartum period (one-time risks)					
	No ARVs	sdNVP	Antenatal ZDV^c^	3-Drug Regimen	Data Sources
ART eligible at conception	0.273 (0.199–0.322)	0.176 (0.082–0.264)	0.136 (0.091–0.157)	0.033 (0.011–0.041)	[24, 55–69]
Non-ART eligible at conception	0.175 (0.127–0.206)	0.073 (0.033–0.109)	0.036 (0.024–0.041)	0.01 (0.004–0.028)	[24, 55–64] [66, 67, 69–71]
Postnatal period (rate per 100 person-years among HIV-uninfected infants aged 4-6 weeks)					
	No ARVs	Extended Infant NVP	3-Drug Regimen	Data Sources	
ART eligible	9.1 (EBF); 15.4 (MBF) (5.7–28.4)	NA	4.0 (0–6.4)	[24, 57, 59, 65, 67, 69–72]	
Non-ART eligible	2.9 (EBF); 4.8 (MBF) (1.8–8.8)	2.7 (1.4–3.7)	2.2 (0–6.4)	[24, 52, 59, 67, 70–77]	
Infant Mortality and Life Expectancy					
Probability of live birth	95.7%–98.0%	MOHCW [21]			
Relative increase in infant mortality if maternal death occurs	2-fold increase	[78–81]			
Short-term mortality risks, %	1-year risk	2-year cumulative risk			
HIV-exposed, uninfected children	7.4 [82]	9.2 [82]			
HIV-infected children, no ART					
Intrauterine/intrapartum infection	51.0 [83]	65.0 [83]			
Postpartum infection	24.0 [83]	38.0 [83]			
HIV-infected children, on ART	9.5 [84]	12.0 [85]			
Life-expectancy estimates, y	Base Case Value	Range for Sensitivity Analyses			
HIV-exposed, uninfected children (from weaning)	50.0 (assumption)	43.0–67.0 [86, 87]			
HIV-infected children, no ART					
Intrauterine/intrapartum infection (from birth)	1.1 [83]	1.1–2.0 (assumption)			
Postpartum infection (from time of infection)	9.4 [83]	5.0–10.0 (assumption)			
HIV-infected children, on ART					
Intrauterine/intrapartum infection (from birth)	20.0 (assumption)	10.0–25.0 (assumption)			
Postpartum infection (from time of infection)	20.0 (assumption)	10.0–25.0 (assumption)			
Maternal Disease Progression Parameters	Value	Data Source			
Impact of antiretroviral therapy					
Efficacy, % HIV RNA suppression at 24 wk					
First-line ART, TDF/FTC + (NVP or EFV)					
Initiated during pregnancy	90%	[88]			
Initiated postpartum, no sdNVP exposure	90%	OCTANE trial [89] Difference: [90–92]			
Initiated postpartum, with sdNVP exposure	85% (difference assumed vs no sdNVP, 5% [88])				
Second-line ART (ZDV/3TC/LPV/r)	72%	[93]			
CD4 cell decline over 6 mo following ART interruption	139 cells/µL	[36–38]			
Laboratory and medication costs	2008 US Dollars	Data Sources			
Economic Model Input Parameters					

CD4 assay, performed once in ANC for Options A, B, and B+	9.42	[33]			
Full blood count, performed once in ANC for Options B and B+	9.27	[94]			
Single-dose NVP, 1 maternal and 1 infant dose	0.06				
Antenatal ZDV, Option A^c^	7.67 per month	[27]			
Antenatal TDF/FTC/NVP, Options B and B+, CD4 count ≤350 cells/µL^c^	12.12 per month	[27]			
Antenatal TDF/FTC/EFV, Options B and B+, CD4 count >350 cells/µL^c^	16.50 per month	[27]			
Postnatal maternal ART					
First-line TDF/FTC/NVP; TDF/FTC/EFV	12.12 per month; 16.50 per month	[27]			
Second line, ZDV/3TC/LPV/r	45.36 per month	[27]			
Pediatric ART, d4T/3TC/NVP	4.54 per month	[27]			
Healthcare Resource Utilization and Costs					
Antenatal care	2008 US Dollars	Data Sources			
Routine antenatal care, 4 visits	45.77	Average of: [95, 96]			
Delivery costs, healthcare facility	54.50	[96]			
Routine and urgent health care costs: Children	No. of Inpatient Days per Year	No. of Outpatient Visits per Year	Total Cost per Month^d^	Data Sources	
HIV-infected children, on ART	2.14	6	3.32	[97]	
Intrauterine/intrapartum infection, no ART	18	6	16.48	[98]	
Postpartum infection, no ART, aged 0–18 mo	18	6	16.48	[98]	
Postpartum infection, no ART, aged >18 mo	11	6	10.67	[98]	
HIV-exposed, uninfected children, aged 0–18 mo	1	3.5	1.73	Assumption^e^	
HIV-exposed, uninfected infants aged >18 mo	0	1	0.26	Assumption^e^	
Terminal care, last month of life	5	0	49.80	Assumption^e^	
Routine and urgent health care costs: Mothers	No. of Inpatient Days per Event	No. of Outpatient Visits per Event	Total Cost per Event^d^	Data Sources	
Care for acute opportunistic infections				Cape Town AIDS Cohort [99]	
WHO stage 3–4 HIV disease, range by specific disease	1.3–2.9	2.7–3.4	21.88–39.36		
Bacterial infection	2.8	2.4	32.28		
Mild fungal infection	1.2	2.3	19.04		
Tuberculosis	2.9	2.2	35.66		
Terminal care, last month of life	2.39	0.77	26.18		
Routine HIV care costs per month	1.22–7.18 (range by CD4)				

See Supplementary Table 2 for complete list of parameters.

Abbreviations: 3TC, lamivudine; ANC, antenatal care; ART, antiretroviral therapy; ARV, antiretroviral medications; d4T, stavudine; EBF, exclusive breastfeeding (in first 6 months of life, followed by MBF); EFV, efavirenz; FTC, emtricabine; HIV, human immunodeficiency virus; LPV/r, lopinavir/ritonavir; MACS, Multicenter AIDS Cohort Study; MBF, mixed breastfeeding; MOHCW, Zimbabwe Ministry of Health and Child Welfare; NA, not applicable; NVP, nevirapine; PTMCT, prevention of mother-to-child HIV transmission; SD, standard deviation; sdNVP, single-dose nevirapine; TDF, tenofovir; WHO, World Health Organization; ZDV, zidovudine.

^a^ ART eligibility was defined as CD4 count of ≤350 cells/µL or WHO stage 3–4 disease.

^b^ PMTCT uptake was defined as proportion of HIV-infected, pregnant women accessing PMTCT services by the time of delivery. See Supplementary Appendix text and Supplementary Table 2 for details.

^c^ Two months of antentatal drug are assumed in all regimens for the base-case analysis, based on median gestational age at booking in Zimbabwe of 30 weeks.

^d^ Total care costs for mothers and infants were calculated by multiplying resource utilization (number of outpatient visits and inpatient days) by an average of WHO-CHOICE estimates of costs for these encounters in 7 sub–Saharan African countries [28]. See Supplementary Appendix for details.

^e^ See Supplementary Table 2 for description of assumptions of outpatient healthcare resource utilization.

#### 

#### Cost Inputs

Monthly medication costs were from the Clinton Healthcare Access Initiative [27]. Costs of clinical care were determined by estimating resource utilization (number of inpatient days and outpatient visits) for specified health conditions, then multiplying by the estimated costs of these healthcare encounters in Zimbabwe (Table 1 and Supplementary Appendix) [28]. For children aged >18 months, monthly utilization estimates (stratified by HIV and ART status) were multiplied by LE to estimate lifetime healthcare costs.

### 

### Model Validation and Sensitivity Analyses

Model-derived risks of MTCT, infant mortality, and postpartum maternal OIs were validated against published data, reported previously with extensive sensitivity analyses [10, 14]. For this study, we conducted univariate and multivariate sensitivity analyses on key PMTCT, pediatric, maternal, and cost parameters.

#### 

#### Access to Care Parameters

We examined the impact of reported rates of PMTCT uptake, defined as the proportion of HIV-infected women receiving PMTCT services and ARVs by delivery (56%, estimated for Zimbabwe in 2009; 80%, the 2009 WHO target goal; 90%, the 2011 WHO target goal; and 95%, reported in neighboring Botswana in 2011) [5, 8, 29]. We varied the availability of CD4 assays from 25% to 100% in Options A, B, and B+; when CD4 count was unavailable in Option A, women were assumed to initiate ART only for WHO stage 3–4 disease. We also examined the impact of reduced pediatric ART availability (36%, estimated for Zimbabwe in 2009) [5] and of reported rates of maternal loss to follow-up (LTFU) from postnatal HIV care (Table 1) [30–32].

#### 

#### Clinical Health Parameters

We defined a lowest-MTCT risk scenario, using the lowest published risks (best reported effectiveness/efficacy) for each modeled regimen (Table 1); a highest-MTCT risk scenario, combining the highest published risks for each regimen; and a scenario assuming equal MTCT risks with Options A and B. We also used 4 assumptions about LE for HIV-exposed and HIV-infected infants: (1) a high pediatric LE scenario, using the upper bound estimates shown in Table 1, (2) a low pediatric LE scenario, using the lower bound estimates, (3) a largest difference scenario (lowest estimates for HIV-infected children; highest estimates for HIV-uninfected children), and (4) a smallest difference scenario (highest estimates for HIV-infected children; lowest estimates for uninfected children).

Finally, we investigated potential maternal health impacts of Option B and B+ in 2 ways. First, we varied the efficacy of first-line ART when resumed after ART interruption, reflecting potential interruption-associated drug resistance. Next, we examined the impact of “treatment fatigue” for women who begin ART with CD4 count >350 cells/µL solely for PMTCT, modeled as (1) an increased risk of virologic failure >6 months after ART initiation or (2) a reduction in second-line ART efficacy.

#### 

#### Cost Parameters

Because estimated costs of healthcare in Zimbabwe are markedly lower than in surrounding countries [28], we repeated the analysis using costs from South Africa (Supplementary Table 2) [33]. In the base case, we conservatively assigned lifelong costs of NVP-based ART to HIV-infected infants; in sensitivity analyses, as an upper bound on pediatric ART costs, we assigned the costs of lifelong lopinavir/ritonavir-based ART to sdNVP-exposed, HIV-infected children. Finally, the nondrug costs of providing 3-drug ARV regimens instead of zidovudine alone (e.g., personnel, laboratory costs) have not been reported; we also examined the impact of such implementation costs in the antenatal period.

## 

## RESULTS

### Base-Case Results

#### Pediatric HIV Risk and LE

Among infants born to HIV-infected women, projected 18-month HIV infection rates were 24.8% (no antenatal ARVs), 14.2% (sdNVP), 7.5% (Option A), and 5.7% (Options B and B+) (Table 2). The resulting projected undiscounted LE (including both HIV-infected and HIV-uninfected infants) ranged from 38.35 years (no antenatal ARVs) to 44.18 years (Options B and B+).

**Table 2.** Base-Case Results: Projected Maternal and Pediatric Outcomes of the Zimbabwe National Prevention of Mother-to-Child HIV Transmission Program

		Pediatric Life Expectancy, Years From Birth		Maternal Life Expectancy, Years From Delivery	
	18-Month Infant HIV Infection Risk	Undiscounted	Discounted	Undiscounted	Discounted
Projected Clinical Outcomes^a^					
No antenatal ARVs^b^	24.8%	38.35	21.34	21.25	14.69
sdNVP	14.2%	41.30	22.45	20.94	14.53
Option A	7.5%	43.27	23.19	21.26	14.70
Option B	5.7%	44.18	23.59	21.30	14.74
Option B+	5.7%	44.18	23.59	22.42	15.45
	Antenatal Care Costs, Through Delivery	Pediatric Lifetime Healthcare Costs, From Birth		Maternal Lifetime HIV-Related Healthcare Costs, From Delivery	
		Undiscounted	Discounted	Undiscounted	Discounted
Projected costs, 2008 US Dollars^a^					

No antenatal ARVs^b^	85	730	520	8490	5280
sdNVP	92	530	360	8460	5300
Option A	118	490	310	8500	5280
Option B	134	370	240	8450	5260
Option B+	134	370	240	9820	6240

Abbreviations: ARVs, antiretroviral medications; HIV, human immunodeficiency virus; sdNVP, single-dose nevirapine.

^a^ Base-case projections assume 100% uptake of PMTCT services by the time of delivery, 100% linkage to HIV care during breastfeeding, no maternal loss to follow-up after delivery, and 100% availability of pediatric antiretroviral therapy (ART) for HIV-infected infants (see Methods).

^b^ No antenatal ARVs refers to receipt of no ARVs or antiretroviral therapy prior to delivery. In all modeled strategies, ART-eligible women who linked to HIV-related healthcare after delivery were assumed to receive ART for their own health in all strategies (Supplementary Table 1).

#### 

#### Pediatric Costs

**Figure 2.**
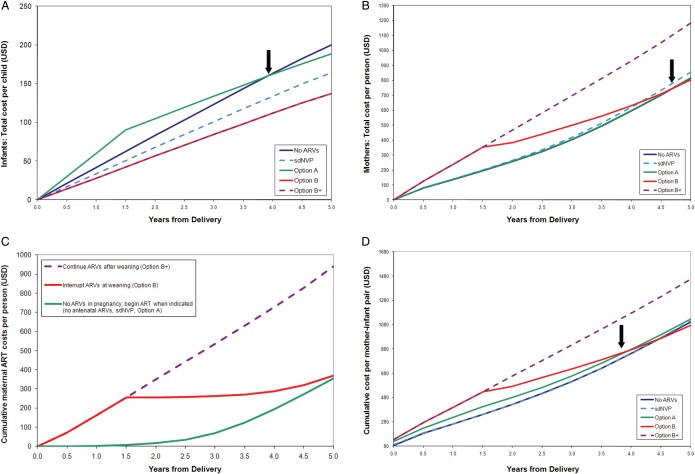
Projected costs (in US dollars [USD]) over the first 5 years after delivery for modeled prevention of mother-to-child human immunodeficiency virus (HIV) transmission (PMTCT) regimens in Zimbabwe. *A*–*D*, Undiscounted costs are shown on the vertical axis, and time from delivery is shown on the horizontal access. *A*, Total healthcare costs for infants (with 100% pediatric antiretroviral therapy [ART] availability). The costs of daily infant nevirapine (NVP) prophylaxis (Option A) are included in pediatric healthcare costs. Because infant NVP is modeled as a pediatric cost, Option A is more expensive than the others during the first 18 months (while breastfeeding continues). PMTCT regimens that are more effective in preventing infant infections result in slower increases in costs (flatter slopes) as time progresses because pediatric HIV care costs are averted, and the pediatric care costs following Option A become less than those following no antenatal antiretroviral medications (ARVs) by 4 years after delivery (arrow). *B*, HIV-related healthcare costs for women after delivery (with 100% retention in care). The costs of maternal ART and 3-drug ARV prophylaxis (Options B and B+) are included in maternal HIV-related healthcare costs. Postnatal care costs are similar following the no antenatal ARVs, single-dose NVP (sdNVP), and Option A strategies: women enrolled in HIV-related care following all 3 of these strategies are assumed to begin ART when CD4 count falls to ≤350 cells/µL or stage 3–4 disease develops. Small cost differences result from assumptions regarding NNRTI resistance following sdNVP, but the slopes of these 3lines are similar. In Option B+, all women continue their 3-drug regimens. In Option B, women who did not have advanced disease before pregnancy interrupt their ARVs but remain in care and re-initiate ART once CD4 count falls to ≤350 cells/µL or stage 3–4 disease develops. As a result, maternal costs after weaning are greater with Option B+ than with the other regimens, and costs for Option B (due to delayed ART use) are much lower after weaning (becoming less than the costs after Option A by 5 years after delivery) (arrow). Antenatal costs are not included in (*A* and *B*). *C*, HIV-infected women with CD4 count >350 cells/µL, post-delivery. ART costs for women not eligible for ART during pregnancy (CD4 count >350 cells/µL, no stage 3–4 disease), from the Cost-effectiveness of Preventing AIDS Complications (CEPAC) adult model. Three postnatal scenarios are shown: (1) initiate 3-drug ARVs in pregnancy and continue ARVs after weaning (as in Option B+); (2) initiate 3-drug ARVs in pregnancy and interrupt ARVs after weaning (Option B); and (3) do not initiate ARVs in pregnancy but remain in care and initiate ART when needed (CD4 count ≤350 cells/µL or stage 3–4 disease, as in the no antenatal ARVs, sdNVP, and Option A strategies). Interrupting ART at weaning saves money compared with continuing ART; however, this ART interruption may be associated with negative health impacts for HIV-infected mothers if retention in care is less than 100% (Table 2). *D*, Total cohort costs over the first 5 years after delivery. These include antenatal care costs (through delivery), maternal HIV-related healthcare costs, and pediatric healthcare costs. Option B becomes cost-saving compared with Option A within 4 years after delivery (arrow). Abbreviations: ART, antiretroviral therapy; ARV, antiretroviral medication; sdNVP, single-dose nevirapine.

PMTCT regimens that prevented more infant infections resulted in lower pediatric healthcare costs over time. After the early cost of infant NVP during breastfeeding, the pediatric healthcare costs of Option A became less than those of no antenatal ARVs by 4 years after delivery (Figure 2*A*). This finding persisted over longer horizons; undiscounted lifetime costs per infant ranged from $730 (no antenatal ARVs) to $370 (Options B and B+) (Table 2).

#### 

#### Maternal LE

Among HIV-infected women, projected undiscounted maternal LE from delivery was 21.25 years (no antenatal ARVs*)*, 21.26 years (Option A*)*, and 22.42 years (Option B+). Projected maternal LE was lowest in the sdNVP strategy (20.94 years, due to the modeled impact of nonnucleoside reverse transcriptase inhibitor resistance on subsequent first-line ART) and intermediate in the Option B strategy (21.30 years, reflecting benefits from ART during pregnancy and breastfeeding but interruption after weaning).

#### 

#### Maternal Costs

Although small differences in short-term maternal costs resulted from modeled drug resistance following sdNVP, 5-year costs were similar for no antenatal ARVs, sdNVP, and Option A (Figure 2*B*). Options B and B+, requiring 3-drug regimens during pregnancy and breastfeeding, conferred the greatest initial maternal healthcare costs. Option B conferred lower maternal costs than Option B+ after weaning because of deferred ART costs when women without advanced disease interrupted ART, and maternal costs with Option B were less than with Option A by 5 years after delivery (Figures 2*B* and 2*C*). Undiscounted lifetime maternal HIV-related costs per woman ranged from $8450 (Option B) to $9820 (Option B+ (Table 2).

### 

### Cost-effectiveness Analysis

Option B was projected to result in a discounted combined lifetime cost (ANC + mother + infant) of $5630 per mother–infant pair and a discounted combined LE (mother + infant) of 38.32 years (Table 3). Compared with Option B, the sdNVP, Option A, and no antenatal ARVs strategies all resulted in lower combined LE (36.03–37.89 years) at greater discounted lifetime costs ($5710–5880 per mother–infant pair) and were therefore “dominated.” Replacing Option B with Option B+ would increase costs ($6620 per mother–infant pair) and LE (39.04 years), with an ICER of $1370 per YLS. Considering total combined costs (ANC + mother + infant), Option B became cost saving compared with Option A by 4 years after delivery (Figure 2*D*; Supplementary Table 7).

**Table 3.** Cost-effectiveness of World Health Organization 2010 Prevention of Mother-to-Child HIV Transmission Guidelines in Zimbabwe

Modeled Scenario and PMTCT Regimen	Combined Costs per Mother–Infant Pair, Discounted, 2008 US Dollars^a^	Combined Life Expectancy per Mother–Infant Pair, Discounted, Years From Delivery^b^	ICER, US Dollars per YLS
Base-Case Projections^c^			
Base-case projections (100% PMTCT uptake, retention in postnatal maternal care, pediatric ART availability)			
Option B	5630	38.32	
Option A	5710	37.89	Dominated^d^
sdNVP	5760	36.97	Dominated
No antenatal ARVs	5880	36.03	Dominated
Option B+	6620	39.04	1370
Sensitivity Analyses^e^			
Access to care parameters:			
Reduced PMTCT uptake (56% of HIV-infected women receiving ARVs by delivery; 87% linkage to postnatal care)			
Option B	4930	35.69	
Option A	4980	35.44	Dominated
sdNVP	5000	34.92	Dominated
No antenatal ARVs	5060	34.39	Dominated
Option B+	5600	36.18	1370
Increased maternal loss to follow-up after delivery (16% in year 1, 6% per year thereafter)			
Option B	3420	35.23	
Option A	3560	34.90	Dominated
sdNVP	3620	34.06	Dominated
No antenatal ARVs	3730	33.05	Dominated
Option B+	3910	35.81	850
Reduced pediatric ART availability (36% of infected children; 2009 Zimbabwe estimate)			
Option B	5610	38.00	
sdNVP	5670	35.96	Dominated
Option A	5670	37.41	Dominated
No antenatal ARVs	5690	34.06	Dominated
Option B+	6590	38.71	1370
Current access to care (56% PMTCT uptake, 87% linkage to postnatal maternal care, increased maternal LTFU, 36% pediatric ART availability)			
Option B	3010	31.99	
sdNVP	3090	30.94	Dominated
Option A	3090	31.72	Dominated
No antenatal ARVs	3100	29.83	Dominated
Option B+	3340	32.38	850
Clinical health parameters:			
“Treatment fatigue”: monthly risk of virologic failure after 6 mo on first-line NNRTI-based ART = 2.39% for women starting ART with CD4 count >350 cells/µL (Options B/B+) (1.5 × base-case risk)			
Option B	5700	37.82	
Option A	5710	37.89	190
sdNVP	5760	36.97	Dominated
No antenatal ARVs	5880	36.03	Dominated
Option B+	6700	38.67	1260
Resource utilization parameters:			
South Africa healthcare costs			
Option B	14 040	38.33	
Option A	14 260	37.89	Dominated
sdNVP	14 730	36.97	Dominated
Option B+	15 070	39.05	1410
No antenatal ARVs	15 520	36.04	Dominated
Additional $150 antenatal implementation cost for 3-drug regimens compared with ZDV alone			
Option A	5760	37.89	
Option B	5760	38.32	2
sdNVP	5770	36.97	Dominated
No ARVs	5880	36.03	Dominated
Option B+	6750	39.04	1370

Abbreviations: ART, antiretroviral therapy; ARV, antiretroviral medications; HIV, human immunodeficiency virus; ICER, incremental cost-effectiveness ratio; LTFU, lost to follow-up; NNRTI, nonnucleoside reverse transcriptase inhibitor; PMTCT, prevention of mother-to-child transmission; sdNVP, single-dose nevirapine; YLS, year of life saved; ZDV, zidovudine.

^a^ Combined costs = PMTCT program costs + maternal lifetime HIV-related healthcare costs + infant lifetime healthcare cost (per mother–infant pair).

^b^ Combined life expectancy = maternal life expectancy from delivery + infant life expectancy from birth.

^c^ Base-case results. Base-case projections assume 100% uptake of PMTCT services by the time of delivery, 100% linkage to HIV care during breastfeeding, no maternal loss to follow-up after delivery, and 100% availability of pediatric ART for HIV-infected infants.

^d^ Dominated refers to an intervention that is more expensive and less effective than an alternative intervention.

^e^ Sensitivity analyses. Please see Supplementary Table 5 for additional details regarding all sensitivity analyses, including the distribution of costs and life expectancy between mothers and infants.

### 

### Sensitivity Analyses

#### Access-to-Care Parameters

The finding that no antenatal ARVs, sdNVP, and Option A were more costly but less effective than Option B was robust with reduced uptake of PMTCT services or access to CD4 testing, as well as with current availability of pediatric ART, and the ICER of Option B+ compared with Option B in these scenarios remained $1370 per YLS (Table 3, Supplementary Table 5). With reported rates of LTFU from maternal postnatal HIV care, the ICER of Option B+ compared with Option B decreased to $850 per YLS. This ICER remained $850 per YLS when current overall access to care in Zimbabwe was simulated (PMTCT uptake, 56%; pediatric ART availability, 36%; maternal LTFU, 16% in year 1, 6% per year thereafter).

#### 

#### Clinical Health Parameters

Base-case policy conclusions were unchanged in all modeled pediatric LE and MTCT risk scenarios, including when MTCT risks were equal with Options A and B, as well as throughout a variety of “treatment fatigue” scenarios for women initiating 3-drug regimens with CD4 count >350 cells/µL (Supplementary Table 5). Results were sensitive, however, to the risk of virologic failure after 6 months on ART. When this risk was increased 1.5-fold from the base case (to >2.4% per month), Option B no longer dominated Option A; when it was increased 2-fold (to 3.2% per month), Option A dominated Option B (Table 3; Supplementary Table 5).

#### 

#### Cost Parameters

Policy conclusions were unchanged when lifelong lopinavir/ritonavir costs were assigned to sdNVP-exposed, HIV-infected infants (Supplementary Table 5). In sensitivity analyses using South Africa healthcare costs, the ICER of Option B+ compared with Option B was $1410 per YLS (Table 3). The difference in antenatal implementation costs between 3-drug regimens and zidovudine alone needed to be ≥$150 per person to change the comparison between Options A and B (Table 3); at $150 per person, Option B was no longer cost saving but remained very cost-effective ($2 per YLS), compared with Option A. Even with implementation costs as high as $400 per person, the ICER of Option B compared with Option A remained <$400 per YLS (Supplementary Table 6).

## 

## DISCUSSION

There are 4 key findings from this work. First, a strategy of providing no antenatal ARVs for PMTCT is more expensive and less effective over a lifetime horizon than strategies based on sdNVP, Option A, or Option B. This result, which occurs because the upfront costs of these PMTCT regimens are greatly outweighed by the downstream costs of caring for HIV-infected infants, lends strong economic support to the well-recognized clinical impact of expanding access to PMTCT programs, regardless of the specific drug regimen provided [5]. Second, in settings where 3-drug ARV regimens are not available for PMTCT [5, 34], replacing sdNVP with Option A benefits infants and mothers and saves money over a lifetime horizon.

Third, healthcare programs would decrease costs and improve outcomes further by implementing Option B instead of Option A. Although short-term drug costs are greater with Option B, the incorporation of healthcare costs for both mothers and infants leads Option B to cost less than Option A within 4 years after delivery, primarily because of averted pediatric HIV costs (Figure 2*D*). Notably, however, if women with high CD4 counts develop poor adherence after Option B (increasing the monthly risk of late virologic failure by ≥25%) (Supplementary Table 5) or if mothers are lost to follow-up after delivery, Option B leads to shorter projected maternal LE than Option A.

Finally, these results strongly support lifelong ART for all pregnant, HIV-infected women (Option B+) [3, 7]. The interruption of effective ART in Option B may have deleterious effects on maternal health. Randomized trial data comparing maternal health outcomes of Options B and B+ are anticipated soon [35]. In the interim, we assume a rapid rate of CD4 decline after ART interruption based on other trials [36–38], with an associated increased risk of OIs. As a result, Option B+ is projected to increase undiscounted maternal LE by 1.12 years compared with Option B (consistent with modeled impacts of other HIV-related interventions [12, 39]), with an ICER of $1370 per YLS. Although this ICER exceeds the 2008 gross domestic product–based threshold for cost-effectiveness in Zimbabwe ($1200 per YLS) [17, 18], it falls in the lower range of ICERs reported for ART-related interventions in developing countries ($550–$5200 per YLS) [19] and thus represents a return on investment comparable with many current HIV programs in Zimbabwe and other resource-limited settings.

Option B+ may represent an even better healthcare investment compared with Option B under specific conditions. First, ART interruption (Option B) may cause greater detriment to maternal health under real-world programmatic conditions than in our guideline-concordant simulations. When women are lost to follow-up after weaning, disease progression is unobserved and cannot lead to prompt ART reinitiation. Such disease progression is more rapid when ART was interrupted months before LTFU (Option B) than at the time of LTFU (Option B+) because of lower CD4 counts at LTFU in Option B. As a result, Option B leads to a projected discounted LE (11.64 years) even lower than no antenatal ARVs (11.71 years) [10], and Option B+ becomes more cost-effective compared with Option B ($850 per YLS). Second, analyses using cost data from South Africa (ICER, $1410 per YLS; 2008 gross domestic product, $5700) [18] suggest that Option B+ may be very cost-effective compared with Option B in higher-income settings where healthcare costs are greater. Third, this analysis excludes several additional benefits of Option B+ that may render it even more effective and cost-effective, including prevention of maternal tuberculosis (also reducing infection risk in infants) [40], HIV transmission to male partners [40], hepatitis B flares due to ARV interruption [7], and MTCT during subsequent pregnancies when women are already on ART at conception [7].

There are several limitations to this analysis. First, all models necessarily simplify complex processes; for example, assumptions about infant LE involved uncertainties about healthcare in the distant future. However, LE assumptions, cost assumptions, and projected clinical and economic results were similar to those previously reported [41, 42], and we tested the impact of biologic and operational assumptions in extensive sensitivity analyses [10, 14]. Except where noted, the impact on policy conclusions was minimal, primarily because assumptions were consistent across PMTCT strategies. Second, we excluded the potential impact of drug-related viral resistance in infants who become infected despite exposure to modeled ARV regimens, because of limited data about acquisition of such resistance [43, 44] and its impact on later ART effectiveness. If resistant HIV is a greater concern for infants who become infected while exposed to maternal ARVs through breastmilk than to extended NVP monoprophylaxis, the benefits of Options B and B+ vs Option A will be attenuated. Finally, our analysis assumed a healthcare system perspective. If a societal perspective were assumed, interventions that avert HIV infections in infants and prevent morbidity and mortality in women would be even more cost-effective, avoiding transportation costs and lost wages for medical care and permitting the productivity gains of healthy women and of children who will become healthy adults.

As in other studies, we find that PMTCT programs based on sdNVP are cost saving, compared with no PMTCT interventions [45]. This is the first analysis to compare sdNVP and Options A, B, and B+ and to consider both short- and long-term maternal and infant outcomes after PMTCT [16, 41, 42, 45]. We find that, with guideline-concordant care, Option A is cost saving compared with sdNVP; Option B becomes more effective and less expensive than Option A within 4 years of delivery; and Option B+ offers additional clinical benefits and economic value comparable with other widely used HIV interventions. We anticipate that the clinical results of these analyses will be generalizable to many African settings where prolonged breastfeeding is the norm and that the base-case economic results may also be applicable in low-income African countries with healthcare costs similar to Zimbabwe. Although specific policies will depend on available resources as well as important considerations of fairness, feasibility, and priority populations [15, 46], PMTCT programs should move rapidly toward these more effective and economically efficient strategies.

## 

## Supplementary Data

Supplementary materials are available at *Clinical Infectious Diseases* online (http://cid.oxfordjournals.org). Supplementary materials consist of data provided by the author that are published to benefit the reader. The posted materials are not copyedited. The contents of all supplementary data are the sole responsibility of the authors. Questions or messages regarding errors should be addressed to the author.

Supplementary Data
