# Supplementary material for: Cost-effectiveness of World Health Organization 2010 Guidelines for Prevention of Mother-to-Child HIV Transmission in Zimbabwe
Source: Clin Infect Dis. 2012 Nov 30;56(3):430–46. doi: 10.1093/cid/cis858 (PMC3540037; doi:10.1093/cid/cis858)
Supplement: Supplementary Data [file supp_56_3_430__index.html]

Cost-effectiveness of World Health Organization 2010 Guidelines for Prevention of Mother-to-Child HIV Transmission in Zimbabwe — Cost-effectiveness of World Health Organization 2010 Guidelines for Prevention of Mother-to-Child HIV Transmission in Zimbabwe — Supplementary Data 

# Cost-effectiveness of World Health Organization 2010 Guidelines for Prevention of Mother-to-Child HIV Transmission in Zimbabwe

## Supplementary Data

Supplementary Data

**Files in this Data Supplement:**

- Supplementary Data - Doc file
