# Supplementary material for: Cost-effectiveness of World Health Organization 2010 Guidelines for Prevention of Mother-to-Child HIV Transmission in Zimbabwe
Source: Clin Infect Dis. 2012 Nov 30;56(3):430–46. doi: 10.1093/cid/cis858 (PMC3540037; doi:10.1093/cid/cis858)
Supplement: Supplementary Data [file supp_cis858_cis858supp.doc]

The Cost-effectiveness of World Health Organization 2010 Guidelines

for Prevention of Mother-to-Child HIV Transmission in Zimbabwe

**Supplemental Appendix**

Andrea L. Ciaranello, *et al.*

**INTRODUCTION**

This appendix is included to provide methodologic details to supplement the description of the methods in the manuscript text, as well as additional model output and results. Similar text regarding model structure and data input parameters has also been included in the Supplemental Appendices of prior publications by the authors (Ciaranello *et al*, *PLoS ONE*, 2011; Ciaranello *et al*, *PLoS Medicine*, 2011) .

**METHODS**

**PMTCT regimens evaluated**

Supplemental Table 1 summarizes the antiretroviral (ARV) regimens recommended in the 2010 World Health Organization guidelines for prevention of mother-to-child transmission (PMTCT) of HIV. Following convention, we refer to combination antiretroviral therapy regimens as “ART” when intended for treatment of maternal HIV disease (also effective for PMTCT) and as "triple-drug ARV regimens" when offered to non-ART-eligible women for PMTCT.

**Uptake of PMTCT services**

To create varied PMTCT uptake scenarios for sensitivity analyses (Supplemental Table 2,

Section 1), the “cascade” of PMTCT services was categorized in three broad domains, consistent with our previous work: “care and testing,” “drug availability,” and “retention" . Uptake rates (proportion of women accessing services) in each domain were derived from best available data, then multiplied to create a total "product of participation" (proportion of women accessing complete services by delivery) to match 2009 Zimbabwean data (56% uptake ), previously and currently WHO-recommended targets (80% and 90% uptake ), and 95% uptake, as reported in neighboring Botswana in 2011.

**Model structure**

The structure of the MTCT, CEPAC adult, and CEPAC infant models details are described below, and the reader is also referred to several key publications providing further detail about model structure and model validation .

*Antenatal and intrapartum outcomes: the MTCT model (Please see also Supplemental Figure 1 and the accompanying legend).*

The MTCT model is a validated simulation model of a cohort of pregnant women, from the time of conception through delivery. A single pregnancy per woman is simulated. The model is a deterministic model, with a decision-tree structure, coded in TreeAgePro software Williamstown, MA) . Key modeled events include the steps in the PMTCT "cascade of care" : presentation to ANC; offer and acceptance of HIV testing; receipt of HIV test results; clinical assessment for ART eligibility; CD4 testing and receipt of results; offer of, acceptance of, and adherence to ARVs for PMTCT; maternal mortality during pregnancy; HIV testing in labor for women with unknown or negative HIV status; live birth; infant HIV infection by the time of delivery; and linkage to postnatal care and ART for mothers and infants.

Probabilities of HIV transmission by 4-6 weeks of age (reflecting *in utero* and intrapartum HIV transmission) are stratified by maternal HIV stage (“ART eligible,” defined as above, or “non-ART-eligible”), and by ARV regimen received for PMTCT. After delivery, model outcomes for infants include vital status and HIV infection/exposure (infected by 4-6 weeks of age or exposed-uninfected); model outcomes for mothers include vital status, CD4 (> or ≤ 350/µL), sdNVP exposure, and current ART receipt. Model validation analyses and key sensitivity analyses have been reported previously .

*Postpartum maternal outcomes: the CEPAC adult model (Please also see Supplemental*

*Figure 2a and the accompanying legend).*

The CEPAC International adult model is a first-order, Monte Carlo simulation of HIV infection in adults. HIV-infected women are simulated individually from delivery through death, and HIV disease progression is characterized by monthly transitions between health states. Health states include acute opportunistic infections (OIs), chronic HIV infection, and death. Monthly risks of OIs and HIV-related death are determined by current CD4 count, OI prophylaxis, and history of previous OIs (Supplemental Table 2, Section IIe). The model records all clinical events and costs accrued monthly over each woman's lifetime; model outcomes include average per-person costs and LE.

Additional structural and technical details of the CEPAC adult model have been reported previously, as have validation analyses comparing model results to published outcomes for postpartum women, as well as men and non-postpartum women . The CEPAC website (http://web2.research.partners.org/cepac) also provides details of model structure, including flow charts depicting patient health states and key modeled events, and protocols for deriving model inputs via regular literature reviews.

*Maternal cohort characteristics and disease progression without ART.* At model entry, women are assigned a baseline HIV RNA level (drawn from the distribution observed in the Cape Town AIDS Cohort, Supplemental Table 2, Section IIa ) and a baseline CD4 cell count (drawn from the distribution observed in the ZVITAMBO trial, Manuscript Table 1). In the absence of effective ART (either before ART initiation or after virologic failure on ART), CD4 counts are modeled to decline at a rate determined by current RNA level (Supplemental Table 2, Section IIe). Current CD4 count, opportunistic infection (OI) prophylaxis, and history or absence of previous OIs determine the monthly risk of OIs and HIV-related death. HIV-related risks of death in the CEPAC model include mortality risks associated with acute opportunistic infection and chronic HIV-infection. Additional risks of death are derived from age- and gender-specific Zimbabwean mortality rates .

*Opportunistic infection prophylaxis.* In addition to antiretroviral therapy, all simulated patients receive trimethoprim-sulfamethoxazole as prophylaxis against *Pneumocystis jiroveci* pneumonia and other bacterial infections, and continue this therapy lifelong . The impact of trimethoprim-sulfamethoxazole prophylaxis on risks of clinical events, including medication toxicities, are described in Supplemental Table 2, Section IIe.

*Antiretroviral therapy.* After delivery, women not yet on ART are modeled to initiate ART when CD4 falls to ≤350/µL or when a WHO Stage 3 or 4 event occurs. With effective ART, modeled HIV RNA suppression leads to CD4 count increases, reducing the monthly risks for OIs and death. Virologic failure on ART may occur either “early” (≤24 weeks) or “late” (>24 weeks) after ART initiation. For patients with early or late virologic failure, CD4 counts decline, accompanied by increased risks of OIs and death. Patients who remain on ART despite virologic failure experience lower risks of OIs and death than do patients who discontinue ART, reflecting the CD4-independent benefit of ART . Patients who interrupt ART while virologically suppressed (*Option B* only, at the time of weaning) experience a rapid decline in CD4 count (mean, 139 cell/µL decline) over the first six months after interruption, based on data from ART interruption and PMTCT trials . Thereafter, CD4 count declines at the “natural history” rate (as before ART was initiated) .

In this analysis, based on 2009 Zimbabwean guidelines, women in HIV-related care were assumed to undergo outpatient clinical evaluation every two months and CD4 monitoring biannually; HIV RNA monitoring was assumed not to be available . ART regimens reflected 2009 Zimbabwean guidelines and common current practice in Zimbabwe, depending on timing of ART initiation (Supplemental Table 2, Section IIe): first-line ART regimens included nevirapine or efavirenz with tenofovir and emtricitabine . Following WHO recommendations, simulated patients were switched from the first to a second antiretroviral regimen after observed clinical or immunologic failure, defined as at least one severe OI, a ≥50% decrease from the peak on-ART CD4 count, or an absolute CD4 count <100 cells/µL . Opportunistic infections diagnosed during the first six months of therapy were not considered as criteria for switching or discontinuing therapy, to allow adequate time for the immunologic benefit of ART to develop. If a second ART regimen was needed, it was comprised of lopinavir/ritonavir with zidovudine/ lamivudine . After failure of 2^nd^-line ART, defined as three severe opportunistic infections or a ≥90% decrease from peak on-ART CD4 count, this 2^nd^-line regimen was continued unless severe toxicity occurred .

*Postnatal outcomes: the CEPAC infant model. (Please also see Supplemental Figure 2b and the accompanying legend).*

The CEPAC infant model is a first-order, Monte Carlo simulation model of infant HIV infection and survival over the first 18 months of life (Supplemental Figure 2b) . For this analysis, infants enter this model at birth and are assigned one of two HIV categories (HIV-exposed but uninfected, or HIV-infected), as well as one of two maternal disease categories (HIV-infected and “ART eligible,” or HIV-infected and “ART non-eligible”). Over the first two years of life, modeled infants face a monthly probability of three key clinical events: 1) maternal death, with risks derived from the adult CEPAC model as above, after which infants are no longer at risk for HIV-infection but are at higher risk of death due to orphanhood ; 2) infant HIV infection through breastfeeding, if infant was previously uninfected; and 3) infant death from any cause. The structure of the CEPAC infant model does not permit changes in maternal ART eligibility or ART use during breastfeeding. MTCT risks are assumed to be constant throughout the period of breastfeeding, based on maternal status at the first postpartum visit, unless maternal death occurs. This slightly underestimates MTCT risk among infants of women with CD4 >350/µL who initially receive the *Option A* regimen (the small proportion of women who have become ART eligible and initiate ART continue to be assigned the MTCT risk of their prior non-ART-eligible status). All infants experience monthly mortality risks and accrue monthly healthcare costs, stratified by infant HIV status, use of ART among infected infants, and maternal vital status. LE estimates (stratified by HIV and ART status) are assigned to infants who survive to postnatal HIV infection or to weaning at 18 months of age (Manuscript Table 1, Section Id).

Detailed, age-stratified data beyond the first several years of life are not readily available regarding mortality among HIV-infected and HIV-uninfected infants and regarding disease progression for HIV-infected infants. Because highly uncertain parameters can lead to wide variability in model results when projected over very long time horizons, we elected not to use the CEPAC infant model to generate life expectancy and lifetime cost projections. However, estimates of life expectancy for HIV-uninfected and HIV-infected infants have been published . For outcomes after 18 months of age in the primary analysis, we therefore added a separate, deterministic, Markov model that could incorporate these life expectancy estimates. The Markov model uses identical health states and transition probabilities to those in the main CEPAC infant model during the first 18 months of life; during this time, life months and healthcare costs are accrued based on time spent in each health state. For infants who become HIV infected or reach 18 months of age without HIV infection, one-time values for remaining life expectancy and remaining lifetime healthcare costs are assigned, stratified by final health state (HIV-exposed, uninfected (assumed in the base-case to have equal life expectancy after weaning to HIV-unexposed infants in Zimbabwe); HIV-infected, without ART; and HIV-infected, treated with ART). Because the Markov model generated only lifetime projections, we used the Monte Carlo model to generate estimates of pediatric healthcare costs over the first five years after birth. For these analyses (Manuscript Figure 2a), we assumed constant mortality rates, based on UNAIDS data , and constant monthly healthcare costs, for children aged 1-5.

**Loss to follow-up in the linked models**

In the base-case analyses, we assumed guideline-concordant care, including 100% retention in care throughout pregnancy, the postpartum period, and lifelong. In sensitivity analyses, we examined the impact of loss to follow-up, to better reflect current programmatic experiences. In the MTCT model, women could be lost to follow-up at any stage between first ANC presentation and delivery . For women lost to follow-up before delivery, no antenatal ARVs were received, but the opportunity to access HIV testing in labor remained. Women could also be lost between delivery and six weeks postpartum or after linkage to postnatal HIV care . In the absence of specific maternal or pediatric data regarding monthly risks of LTFU and cessation of prophylactic ARVs during breastfeeding, the impact of such events was incorporated in sensitivity analyses via the “highest risk” postnatal transmission estimates for *Options A, B,* and *B+.*

**Linkages between the models**

The MTCT and CEPAC models were linked to allow a combined analysis in which each woman-infant pair is simulated together from the time of first presentation at ANC through pregnancy and delivery (the MTCT model), and then each woman and infant are simulated separately throughout their lifetimes (the CEPAC models). This linkage was accomplished by first simulating maternal and infant outcomes in the appropriate CEPAC models, then using CEPAC model results (postnatal infection risk, LE, and costs) as inputs to the MTCT model (Supplemental Figure 1).

**Sensitivity analyses**

Because of their varied structures, each of the models used in this analysis permits different techniques for the evaluation of uncertainty (for example, Markov models and decision trees permit probabilistic sensitivity analyses, while Monte Carlo models do not readily facilitate this methodology). In order to apply consistent methods for conducting sensitivity analyses among all of the linked models, and to increase transparency and comprehensibility for the reader, we followed the guidance of the US Panel on Cost-effectiveness in Health and Medicine and conducted extensive univariate and multivariate sensitivity analyses on model parameters shown to be influential in prior work and considered to be potentially influential for this analysis.

**Model input data**

*MTCT risks (Manuscript Table 1 and Supplemental Table 2, Section IIc).* MTCT risks for each modeled regimen are shown in manuscript Table 1 and are derived from key PMTCT studies in breastfeeding populations in Africa . In the absence of reported postnatal transmission risks for infants older than six months of age who continue to breastfeed with ongoing prophylaxis (*Option A* or *B*), we assumed a constant monthly risk based on data in younger children, as has been observed for postnatal transmission without prophylaxis . MTCT risks during the intrauterine and intrapartum period (by 4-6 weeks of age) and postpartum period (6 weeks-18 months) were stratified by maternal HIV stage and by PMTCT regimen received. Postpartum transmission risks were additionally stratified by whether breastfeeding during the first six months of life was exclusive (EBF) or mixed (MBF, including any non-breastmilk liquid or solid) .

*Infant mortality rates and life expectancy estimates (**Manuscript Table 1 and Supplemental Table 2, Section IId).* Mortality for HIV-exposed, uninfected infants was from the ZVITAMBO study in Zimbabwe , and mortality for HIV-infected infants was from pooled analyses of several African cohorts (Table 1, Section Id) . Maternal death was assumed to increase infant mortality in the first 18 months of life 2-fold . LE after 18 months for HIV-uninfected infants was assumed to be 50 years, based on estimates ranging from 43-67 years . LE estimates for untreated infants with intrauterine/intrapartum (1.1 years) or postpartum (9.4 years) HIV infection were from pooled UNAIDS data , and LE for HIV-infected children on ART was assumed to be 20 years.

*Economic data inputs (Manuscript Table 1 and Supplemental Table 2, Section III).* In order to assign costs to each health state or clinical encounter in the computer models, we undertook a two-step approach. Step 1 involved estimating the healthcare resource utilization required for each health state or clinical encounter (for example, number of outpatient visits required annually for routine HIV care, number of inpatient days required for treatment of acute *Pneumocystis jiroveci* infection, etc). These resource utilization estimates were taken from available programmatic and published data, as outlined in Supplemental Table 2. Step 2 involved assigning a cost, in 2008 US dollars (USD), to each health state or encounter.

In most settings, Step 2 can be accomplished by multiplying resource utilization (e.g number of inpatient days) by WHO-CHOICE estimates of the cost per inpatient day (“unit cost”) . However, estimating the unit cost for health care utilization in Zimbabwe is complicated by large currency fluctuations and changes in monetary policy in the recent past. The Zimbabwe currency was devalued in 2006, 2008, and 2009, with an official inflation rate of 231,000,000% reported for 2008 . This poses a major challenge in terms of effectively updating costs collected in Zimbabwe in prior years. (For example, the official exchange rate (OER) of Zimbabwean dollars to USD was reported at 0.6788 to 1 in 1978 and ~500,000 to 1 in 2006. When a second Zimbabwean dollar was created in 2006, the OER was 250-550 Zimbabwean dollars to 1 US$. In July 2008 this second dollar was converted at 10,000,000,000 to 1 new Zimbabwean dollar, and then in February 2009, converted again at 1,000,000,000,000 to 1 Zimbabwean dollar; the Zimbabwean dollar was then officially abandoned in April 2009 as a currency). Currently, the USD is the official currency in Zimbabwe, with unofficial transactions also occurring in foreign currencies such as the Euro, South African Rand, Botswana Pula, and Pound Sterling.

Because of this hyperinflation, the most recent year in which gross national income (GNI) per capita was reported for Zimbabwe by the World Bank (World Development Indicators) was 2005. In that year, GNI per capita was 360 USD, a sharp decline from the previous two years ($790 in 2003, $590 in 2004) . Although the WHO-CHOICE data reports unit cost estimates for outpatient visits and hospital stays in Zimbabwe in 2005 International dollars (I$), it is not possible to convert these estimates to current USD, since the year the data was collected, and the then-prevailing exchange rate, is not known. Currently, USD and other foreign currencies, such as South African Rand, are used for market transactions in Zimbabwe, but no data were available regarding the current prices for health services purchased with foreign currencies at the time of this analysis. In lieu of current data on the cost of health services in Zimbabwe, we used data from six low-income, heavily HIV-affected countries in sub-Saharan Africa with similar GNI per capita. These included Malawi (GNI per-capita: $210 in 2005, $280 in 2009 in USD), Mozambique (GNI per-capita: $290 in 2005, $440 in 2009), Uganda GNI per-capita: $300 in 2005, $460 in 2009), Tanzania (GNI per-capita: $350 in 2005, $500 in 2009), Zambia (GNI per-capita: $500 in 2005, $970 in 2009), and Kenya (GNI per-capita: $520 in 2005, $770 in 2009) . For each of these countries, we obtained 2005 WHO-CHOICE estimates of unit costs, and converted them into 2008 USD. We took the average of the six countries and used this as our estimate of unit costs for Zimbabwe (Supplemental Table 3).

In the absence of primary data from Zimbabwe to inform the cost of PMTCT- and HIV-related services, and because we cannot convert WHO-CHOICE 2005 estimates for Zimbabwe from I$ to USD due to hyperinflation, we feel comfortable that that the derived estimates in USD are reasonable. For example, the original 2005 WHO-CHOICE estimates for Zimbabwe in I$ are 30.77 per tertiary inpatient bed-day, and 10.02 per outpatient visit. These estimates fall within the bounds of these same estimates in I$ for the six countries used to derive the average estimate (Supplemental Table 3: $12.06 – 34.99 per inpatient day; $3.35 – 11.65 per outpatient visit).

**RESULTS**

**Intermediate outcomes: CEPAC Model Results (Supplemental Table 4)**

Selected results of the CEPAC adult and CEPAC infant models are shown in Supplemental

Table 4; results shown are undiscounted. Discounted and undiscounted life expectancies and per-person HIV-related healthcare costs were generated for key categories of mothers and infants, and used as inputs to the MTCT model.

**Sensitivity analyses (Manuscript Table 3 and Supplemental Tables 5 and 6)**

Results of the cost-effectiveness analyses for the base case and key sensitivity analyses are shown in Supplemental Table 5. Key components of each analysis are shown, including antenatal care costs/mother-infant pair, pediatric lifetime healthcare costs/person, maternal lifetime HIV-related healthcare costs/person, pediatric life expectancy from birth, and maternal life expectancy from delivery.

In the absence of data to inform the costs required to provide three-drug ARV regimens, such as healthcare center infrastructure, personnel, and laboratory monitoring, Supplemental Table 6 shows the impact of assumptions about these costs. In the base-case analysis, the difference in cost between the antenatal ZDV regimen and three-drug ARV regimens is $25 (this includes drug costs, as well as a single full blood count). Supplemental Table 6 shows that the comparison of *sdNVP* to *no antenatal ARVs* does not change as the cost of implementing 3-drug ART is increased to at least $500 per patient. Similarly, the comparison between *Option B* and *Option B+* (both of which provide 3-drug ART regimens during pregnancy) also does not change. In order to change the comparison between *Option A* and *sdNVP*, the cost of implementing 3-drug regimens must be at least $200 per patient; above $200/patient. *Option A* no longer dominates *sdNVP*, but remains very cost-effective compared to *sdNVP*. In order to change the comparison between *Option A* and *no antenatal ARVs*, implementation of 3-drug ART must be between $400 and $500/patient; at $500, *Option A* no longer dominates *no antenatal ARVs*, but remains very cost-effective compared to *sdNVP*. In order to change the comparison between *Option A* and *Option B*, the implementation cost of 3-drug regimens must exceed **$150**/patient; between **$150** and nearly $500/patient, *Option B* no longer saves money compared to (dominates) *Option A*, but remains very cost-effective compared to *Option A*.

**Cumulative short-term costs (first five years after delivery; Manuscript Figures 2a-d and Supplemental Table 7).**

Supplemental Table 7 shows the cumulative costs for mothers and infants of the modeled PMTCT strategies. The results listed include ANC costs (through delivery) and maternal and pediatric costs from 1-5 years after delivery. These results are also shown in graphical form in Manuscript Figures 2a-2d. ART costs for a subset of HIV-infected women after delivery are shown in Manuscript Figure 2c and Supplemental Figure 3.

**ADDITIONAL NOTES: COMPARISON OF CEPAC AND UNAIDS/SPECTRUM ESTIMATES OF MTCT RISK**

We have formally compared the MTCT risk input parameters derived in our analysis with the MTCT risks derived by the UNAIDS/Spectrum Reference Group on Estimates, Modelling and Projections . These comparisons are shown in Supplemental Table 8.

Supplemental Table 1. PMTCT regimens simulated in a computer model of PMTCT services in Zimbabwe

| **PMTCT**  **Regimen** | **Antenatal**  **(maternal)** | **Intrapartum**  **(maternal)** | **Postpartum**  **(maternal)** ^a^ | **Neonatal**  **(infant)** |
| --- | --- | --- | --- | --- |
| ***No antenatal ARVs*** | None | None | Stage 3-4 disease: d4T/3TC/NVP | None |
| ***sdNVP*** | Stage 3-4 disease: ZDV/3TC/NVP from ≥28 weeks  Others: None | Stage 3-4 disease: ZDV/3TC/NVP  Others: sdNVP | Stage 3-4 disease: ZDV/3TC/NVP | sdNVP |
|  |  |  |  |  |
| ***WHO “Option A”*** | *CD4≤350/µL or Stage 3-4 disease:* ZDV/3TC/NVP from ≥14 weeks or first ANC visit | *CD4≤350/µL or Stage 3-4 disease:* ZDV/3TC/NVP | *CD4≤350/µL or Stage 3-4 disease:* ZDV/3TC/NVP through breastfeeding | *CD4≤350/µL or Stage 3-4 disease:* NVP x 6 weeks |
|  | *CD4>350/µL and Stage 1-2 disease:*  ZDV from ≥14 weeks or first ANC visit | *CD4>350/µL and Stage 1-2 disease:*  sdNVP  + ZDV/3TC ^b^ | *CD4>350/µL and Stage 1-2 disease:*  ZDV/3TC x 7days* | *CD4>350/µL and Stage 1-2 disease:*  NVP through BF |
| ***WHO “Option B”*** | Regardless of CD4: ZDV/3TC/NVP from ≥14 weeks or first ANC visit | Regardless of CD4: ZDV/3TC/NVP | Regardless of CD4: ZDV/3TC/NVP through breastfeeding | Regardless of CD4:  NVP x 6 weeks |
|  |  |  |  |  |
| ***“Option B+”*** | Regardless of CD4: ZDV/3TC/NVP from ≥14 weeks or first ANC visit | Regardless of CD4: ZDV/3TC/NVP | Regardless of CD4: ZDV/3TC/NVP, continued lifelong | Regardless of CD4:  NVP x 6 weeks |

**ARVs:** antiretroviral drugs; **sdNVP**: single-dose nevirapine; NPV: nevirapine; **ZDV**: zidovudine; **3TC**: lamivudine, **BF**: breastfeeding.

**a.** Per 2010 WHO guidelines, sdNVP and intra/postpartum ZDV/3TC can be omitted if the mother receives >4 weeks of ZDV before delivery .

**b.** The 2010 WHO PMTCT guidelines do not specifically address postnatal regimens for women who present late to antenatal care, and therefore receive only sdNVP as the antenatal/intrapartum component of their PMTCT regimens. In the “sdNVP” regimen, we assumed that women would not receive any postpartum medications (such as a ZDV/3TC tail or any extended postnatal prophylaxis), to reflect the 2002-2009 national PMTCT program. In the WHO 2010 guidelines regimens (*Options A* and *B*), we assumed that such women would link to postnatal HIV care at a rate based on published data. Women in postnatal HIV care would then undergo CD4 testing, and would initiate ART if CD4 ≤350/µL or WHO stage 3-4 disease was evident. Women found to have CD4 counts >350/µL would receive three-drug ARVs for breastfeeding prophylaxis (*Option B*) or their infants would receive extended NVP prophylaxis (*Option A*).

**Supplemental Table 2. Complete input parameters for a model of mother-to-child transmission in Zimbabwe (includes parameters listed in manuscript Table 1)**

| **I. PMTCT coverage scenarios** | | | | | | | |
| --- | --- | --- | --- | --- | --- | --- | --- |
| **Scenario** | **Care and testing ^a^** | **Drug availability ^b^** | | **Retention in care by delivery ^c^** | | **Product of participation by delivery ^d^** | |
| **Zimbabwe 2009 (56%)** | ANC: 91%  HIV testing: 87%  Result return: 99%  Total: 78% (=91%*87%*99%) | 82% | | 87% | | To delivery: 56% | |
| **2009 WHO target (80%)** | ANC: 100%  HIV testing: 80%  Result return: 100%  Total: 80% (=100%*80%*100%) | 100% | | 100% | | To delivery: 80% | |
| **2011 WHO target (90%) Uptake** | ANC: 100%  HIV testing: 90%  Result return: 100%  Total: 90% (=100%*90%*100%) | 100% | | 100% | | To delivery: 90% | |
| **Optimal (95%) Uptake** | ANC: 100%  HIV testing: 95%  Result return: 100%  Total: 95% (=100%*95%*100%) | 100% | | 100% | | To delivery: 95% | |
| **II. Clinical model input parameters** | | | | | | | |
| **IIa. Baseline maternal cohort characteristics** | | | | | | | |
| **Variable** | | | **Base Case Value** | |  | | **Data sources** |
| Age (mean (SD), years) | | | 24 (5) | |  | | MOHCW |
| Mortality during pregnancy | | | 0.7% | |  | | MOHCW |
| Proportion ART-eligible^e^ | | | 36% | |  | | ZVITAMBO trial |
| CD4 count (mean (SD), /µL) | | |  | |  | |  |
| Total cohort | | | 451 (50) | |  | | ZVITAMBO trial |
| ART-eligible women | | | 275 (50) | |  | | ZVITAMBO trial |
| Non-ART-eligible women | | | 550 (50) | |  | | ZVITAMBO trial |

Supplemental Table 2, continued.

| **II. Clinical model input parameters, continued** | | | | | | | |
| --- | --- | --- | --- | --- | --- | --- | --- |
| **IIa. Baseline maternal cohort characteristics, continued** | | | | | | | |
| **Variable** | | | **Base Case Value** | | | **Data source** | |
| Distribution of initial HIV RNA (% total) | | |  | | | Cape Town AIDS Cohort | |
| >100,000 copies/ml | | | 43 | | |  | |
| 30,001-100,000 copies/ml | | | 28 | | |  | |
| 10,001-30,000 copies/ml | | | 18 | | |  | |
| 3,001-10,000 copies/ml | | | 8 | | |  | |
| 501-3,000 copies/ml | | | 2 | | |  | |
| ≤ 500 copies/ml | | | 1 | | |  | |
| **IIb. Uptake of PMTCT services and postnatal care (equal for all coverage scenarios)** | | | | | | | |
| **Variable** | | | **Base Case Value** | | **Data sources** | | |
| Sensitivity of clinical assessment of ART eligibility ^e^ | | | 36% | | MTCT-Plus Cohort | | |
| Delivery in health care facility | | | 69% | | Zimbabwe MOHCW | | |
| Probability of linking to pediatric HIV diagnosis, care, and ART | | | 100% (sensitivity analysis: 36%) | | WHO/UNICEF | | |
| Duration of breastfeeding (months) | | | 18 | | WHO, ZVITAMBO trial | | |
| Probability of linking to postnatal maternal HIV-related care | | | 100% | |  | | |
| If antenatal care received | | | (Sensitivity analysis: 87%) | | Mean of | | |
| If no antenatal care received | | | (Sensitivity Analysis: 43%) | | Assumption (50% of above) | | |
| Loss to follow-up from postnatal maternal care | | | 0%/year  Sensitivity analyses: 16% (year 1);  6%/year (years 2+) | |  | | |
| **IIc. Mother-to-child transmission risks** | | | | | | | |
|  | **Base Case Value (Range for sensitivity analysis)** | | | | | | |
| **Maternal HIV status** | **PMTCT regimen received** | | | | | | |
| **Intrauterine/intrapartum period (one-time risks)** | | | | | | | |
|  | | **No ARVs** | **sdNVP** | **Antenatal ZDV^f^** | | | **Triple-drug regimen** |
| ART-eligible at conception | | 0.273  (0.199-0.322) | 0.176  (0.082-0.264) | 0.136  (0.091-0.157) | | | 0.033  (0.011-0.041) |
| Non-ART-eligible at conception | | 0.175  (0.127-0.206) | 0.073  (0.033-0.109) | 0.036  (0.024-0.041) | | | 0.01  (0.004-0.028) |

Supplemental Table 2, continued.

| **IIc. Mother-to-child transmission risks, continued.** | | | | | | | |
| --- | --- | --- | --- | --- | --- | --- | --- |
|  | | **Base Case Value (Range for sensitivity analysis)** | | | | | |
| **Maternal HIV status** | | **PMTCT regimen received** | | | | | |
| **Postnatal period (rate/100 person-years, among infants HIV-uninfected at 4-6 weeks of age)** | | | | | | | |
|  | **No ARVs** | | | **Extended infant NVP** | | **Triple-drug regimen** | |
| ART-eligible | 9.13 (EBF) ; 15.43 (MBF)  (5.73-28.36) | | | n/a | | 4.00  (0-6.42) | |
| Non-ART-eligible | 2.86 (EBF) ; 4.82 (MBF)  (1.79-8.82) | | | 2.65  (1.44-3.74) | | 2.23  (0-6.42) | |
| **IId. Infant mortality and life expectancy** | | |  | | | |  |
| **Variable** | | | **Base Case Value** | | | | **Data sources** |
| Probability of live birth | | | 95.7-98% | | | | MOHCW |
| Relative increase in infant mortality if maternal death occurs | | | 2-fold increase | | | |  |
| **Short-term mortality risks:** | | | **One-year risk (%)** | | **Two-year cumulative**  **risk (%)** | |  |
| HIV-exposed, uninfected children | | | 7.40 | | 9.20 | |  |
| HIV-infected children, no ART | | |  | | | | |
| Intrauterine/intrapartum infection | | | 51.0 | | 65.0 | |  |
| Postpartum infection | | | 24.0 | | 38.0 | |  |
| HIV-infected children, on ART | | | 9.5 | | 12.0 | |  |
| **Life-expectancy estimates** | | | **Base Case Value (years)** | | **Range for sensitivity analyses (years)** | | **Data sources** |
| HIV-exposed, uninfected children (from weaning) | | | 50.0 | | 43.0-67.0 | |  |
| HIV-infected children, no ART | | |  | |  | |  |
| Intrauterine/intrapartum infection (from birth) | | | 1.1 | | 1.1-2.0 | | , assumption |
| Postpartum infection (from time of infection) | | | 9.4 | | 5.0-10.0 | | , assumption |
| HIV-infected children, on ART | | |  | |  | |  |
| Intrauterine/intrapartum infection (from birth) | | | 20.0 | | 10.0-25.0 | | Assumption |
| Postpartum infection (from time of infection) | | | 20.0 | | 10.0-25.0 | | Assumption |

Supplemental Table 2, continued.

| **IIe. Maternal disease progression parameters** |  | |  |
| --- | --- | --- | --- |
| **Natural history (in absence of antiretroviral therapy)** |  | | **Data Sources** |
| Mean monthly decrease in CD4/µL by HIV RNA |  | | Multicenter AIDS Cohort Study |
| >30,000 copies/ml | 6.4 | |  |
| 10,001-30,000 copies/ml | 5.4 | |  |
| 3,001-10,000 copies/ml | 4.6 | |  |
| 501-3,000 copies/ml | 3.7 | |  |
| 0-500 copies/ml | 3.0 | |  |
| Monthly risk of severe opportunistic infections (%, range by CD4 count) | | | Cape Town AIDS Cohort |
| WHO stage III-IV |  | |  |
| Visceral | 0.00-1.52 | |  |
| Non-visceral | 0.02-2.26 | |  |
| Non-specific | 0.00-0.71 | |  |
| Bacterial infection | 0.03-0.71 | |  |
| Tuberculosis | 0.16-1.96 | |  |
| Other severe infection | 0.14-1.67 | |  |
| Monthly risk of other clinical conditions (%, range by CD4 count) | | | Cape Town AIDS Cohort |
| Mild fungal infection | 1.76-3.14 | |  |
| Other mild infection | 2.33-2.67 | |  |
| Monthly risk of death from severe opportunistic infection (%) |  |  | Cape Town AIDS Cohort |
| WHO stage III-IV |  | |  |
| Visceral | 9.21 | |  |
| Non-visceral | 2.38 | |  |
| Non-specific | 20.00 | |  |
| Bacterial infection | 2.94 | |  |
| Tuberculosis | 1.82 | |  |
| Other severe infection | 6.67 | |  |

Supplemental Table 2, continued.

| **IIe. Maternal disease progression parameters, continued** |  |  |
| --- | --- | --- |
| **Natural history (in absence of antiretroviral therapy), continued** | | **Data Sources** |
| Monthly risk of death from other clinical conditions (%) |  | Cape Town AIDS Cohort |
| Mild fungal infection | 0.54 |  |
| Other mild infection | 0.39 |  |
| Monthly risk of HIV-related death (%, range by CD4 count) |  | Cape Town AIDS Cohort |
| No history of opportunistic infection | 0.00-4.02 |  |
| With history of opportunistic infection | 0.00-9.53 |  |
| Relative risk reduction on any ART regimen (%, range by CD4) |  | Cotrimo-CI, ANRS 1203 |
| HIV-related death | 55-96 |  |
| Acute opportunistic infections | 0-32 |  |
| **Impact of trimethoprim-sulfamethoxazole prophylaxis** |  |  |
| % Reduction in probability of infection |  |  |
| Mild bacterial diseases | 48.79 | Cotrimo-CI |
| Invasive bacterial diseases | 49.81 |  |
| WHO stage III-IV visceral diseases and other severe events | 17.88 |  |
| Toxicity of trimethoprim-sulfamethoxazole (%, one-time risk) |  | Cotrimo-CI |
| Minor toxicity | 18.24 |  |
| Major toxicity | 6.72 |  |
| **Impact of antiretroviral therapy** | |  |
| Efficacy (% HIV RNA suppression at 24 weeks) ; gain in CD4/µL at 24 weeks on suppressive ART; yearly risk (%) of virologic failure >24 weeks after initiation | |  |
| 1^st^-line ART (TDF/FTC + (NVP or EFV)) |  |  |
| Initiated during pregnancy | 90%; 148;17.45% |  |
| Initiated postpartum, no sdNVP exposure (or if sdNVP followed antenatal ZDV) | 90%; 148;17.45% | OCTANE trial ; |
| Initiated postpartum, with sdNVP exposure | 85%  Difference assumed = 5% |  |
| 2^nd^ line ART (ZDV/3TC/LPV/r) | 72%; 148;17.45% |  |
| CD4 cell decline over 6 months following ART interruption | 139 cells/µL |  |

Supplemental Table 2, continued.

| **III. Economic model input parameters** | | | |
| --- | --- | --- | --- |
| **IIIa. Laboratory and medication costs** | **2008 USD** | | **Data sources** |
| CD4 assay (performed once in ANC for *Options A, B,* and *B+)* | 9.42 | |  |
| Full blood count (peformed once in ANC for *Options B* and *B+)* | 9.27 | |  |
| Single-dose nevirapine (1 maternal and 1 infant dose) | 0.06 | |  |
| Antenatal zidovudine (*Option A)* | 7.67/month | | (base-case: 2 months antenatal drug)*** |
| Antenatal TDF/FTC/NVP (*Options B* and *B+*, CD4 >350/µL) | 12.12/month | | (base-case: 2 months antenatal drug)*** |
| Antenatal TDF/FTC/EFV (*Options B* and *B+,* CD4 ≤350/µL) | 16.50/month | | (base-case: 2 months antenatal drug)*** |
| Postnatal maternal ART |  | |  |
| 1st-line (TDF/FTC/NVP) | 12.12/month | |  |
| 2nd line (ZDV/3TC/LPV/r) | 45.36/month | |  |
| Pediatric ART (d4T/3TC/NVP) | 4.54/month | |  |
| Cotrimoxazole  --Modeled as received by all HIV-infected children, 54% of HIV-exposed children, and all HIV-infected mothers in postnatal care with CD4 ≤350/µL | 1.02 | | Gauteng Hospitals |
| **IIIb. Antenatal care utilization and costs** | **2008 USD** | | **Notes and data sources** |
| Routine antenatal care (4 visits) | 45.77 |  | Average of: |
| HIV testing in ANC |  |  |  |
| Test kit | 1.20 |  | Kit cost: average of ; 1.16 kits/person, based on 2008 testing algorithm, 99% sensitivity, 99% specificity |
| 10 minutes nurse time (pretest counseling) | 0.17 |  | Time: assumption; Salary $1500/year |
| 25 minutes nurse time (posttest counseling, positive result) | 0.44 |  | Time: ; Salary $1500/year |
| 15 minutes nurse time (posttest counseling, negative result) | 0.26 |  | Time: assumption; Salary $1500/year |
| Delivery costs (healthcare facility) | 54.50 |  |  |

Supplemental Table 2, continued.

| **III. Economic model input parameters, continued** | | | | | | | |
| --- | --- | --- | --- | --- | --- | --- | --- |
| **IIIc. Pediatric healthcare utilization and costs** | **# Inpatient days/year** | | **# Outpatient visits/year** | | **Total cost/month** | | |
| **Ongoing healthcare costs** |  | |  | | Zimbabwe  (base-case) | South Africa (sensitivity analysis) | |
| HIV-infected children, on ART | 2.14 | | 6 (assumed 2-monthly) | | 3.32 | 44.71 | |
| Intrauterine/intrapartum infection, no ART | 18 (high estimate) | | 6 (assumed 2-monthly) | | 16.48 | 336.36 | |
| Postpartum infection, no ART (age 0-18m) | 18 (high estimate) | | 6 (assumed 2-monthly) | | 16.48 | 336.36 | |
| Postpartum infection, no ART (age >18m) | 11 (average estimate) | | 6 (assumed 2-monthly) | | 10.67 | 207.64 | |
| HIV-exposed, uninfected children (age 0-18m) | 1 (assumption) | | 3.5 (4 routine vaccination visits + 3 urgent visits over 2 years) | | 1.73 | 21.51 | |
| HIV-exposed, uninfected infants (age >18m) | 0 (assumption) | | 1 (assumption) | | 0.26 | 0.89 | |
| **Terminal care, last month of life** | 5 (assumption) | | 0 (assumption) | | 49.80 | 1103.36 | |
| **IIId. Maternal healthcare utilization and costs** | **# Inpatient days** | **# Outpatient visits** | | **Total cost/event** | | | **Data source** |
| **Acute clinical event Costs (per event, range by OI type)** |  |  | | Zimbabwe  (base-case) | South Africa (sensitivity analysis) | | Resource utilization: , costs: see Methods above |
| Care for acute OI (per event) |  |  | |  |  | |
| WHO stage III-IV |  |  | |  |  | |
| Visceral | **2.9** | **3.4** | | **39.36** | 685 | |
| Non-visceral | **1.8** | **2.7** | | **26.24** | 432 | |
| Non-specific | **1.3** | **2.9** | | **21.88** | 322 | |
| Bacterial infection | **2.8** | **2.4** | | **32.28** | 652 | |
| Mild fungal infection | **1.2** | **2.3** | | **19.04** | 293 | |
| Tuberculosis | **2.9** | **2.2** | | **35.66** | 672 | |
| Other mild infection | **0.7** | **2.2** | | **13.75** | 180 | |
| Other severe infection | **1.8** | **2.6** | | **25.94** | 430 | |
| Terminal care, last month of life | 2.39 | 0.77 | | 26.18 | 543 | |  |

Supplemental Table 2, continued.

| **III. Economic model input parameters, continued** | | | | | |
| --- | --- | --- | --- | --- | --- |
| **IIId. Maternal healthcare utilization and costs, continued** | **# Inpatient days** | **# Outpatient visits** | **Total cost** | | **Data source** |
| **Routine Care Costs (per month)** |  |  | Zimbabwe  (base-case) | South Africa (sensitivity analysis) |  |
| >500 | 0.03 | 0.3 | 1.22 | 9.96 | Resource utilization: , costs: see Methods above |
| 351-500 | 0.06 | 0.27 | 1.43 | 16.34 |
| 201-350 | 0.08 | 0.29 | 1.69 | 21.03 |
| 51-200 | 0.22 | 0.29 | 3.08 | 52.32 |
| <50 | 0.56 | 0.52 | 7.18 | 130.80 |
|  |  |  |  | |  |
| **Medication toxicity (per event): ART or cotrimoxazole** |  |  |  | |  |
| Minor/moderate toxicity | 0 | 1 | 3.08 | 10.84 | Cape Town AIDS Cohort, Health Systems Trust |
| Major/severe toxicity | 7 | 0 | 69.72 | 1564.52 | Cape Town AIDS Cohort, Health Systems Trust |

**SD**: Standard deviation; **MOHCW:** Zimbabwe Ministry of Health and Child Welfare; **ART**: antiretroviral therapy; **ANC**: antenatal care; **sdNVP**: single-dose nevirapine; **WHO**: World Health Organization; **NVP**: nevirapine; **ZDV**: zidovudine; **3TC**: lamivudine; **d4T**: stavudine; **LPV/r**: lopinavir/ritonavir; **TDF**: tenofovir; **FTC**: emtricabine; **EFV**: efavirenz; **MACS**: Multicenter AIDS Cohort Study; **EBF**: exclusive breastfeeding (in first six months of life, followed by MBF); **MBF**: mixed breastfeeding.

**PMTCT uptake scenarios:**

**a. Care and testing**: Proportion of pregnant women accessing antenatal care, HIV testing for those in antenatal care, and receipt of HIV test result for those tested.

**b. Drug availability**: Proportion of antenatal care sites with access to medications for PMTCT. This proportion is back-calculated in order to reach the reported product of participation for each scenario.

**c. Retention**: Of women offered ARVs for PMTCT, the proportion remaining in care during the antenatal period, used as a proxy for acceptance of and adherence to medications.

**d. Product of participation**: Proportion of patients receiving care at all stages of the PMTCT cascade, defined as the product of (drug availability)*(care and testing)*(retention).

**Supplemental Table 2, continued (footnotes).**

**Other notes:**

**e. ART eligibility**. Defined as CD4 ≤350/µL or WHO Stage 3-4 disease In scenarios in which CD4 assays were not available, we simulate clinical assessment of ART-eligibility. The sensitivity of clinical assessment of ART eligibility was reported for a CD4 threshold of 200/µL (36%); model sensitivity analyses using subsequent reports based on a CD4 threshold of 350/µL (sensitivity: 20%) did not substantially change results in prior work .

**f. Antenatal ZDV:** This reflects the antenatal component of the *Option A* regimen for women who are not eligible for ART. Per WHO 2010 PMTCT guidelines, the intrapartum sdNVP and 7-day postnatal ZDV/3TC "tail" components of the *Option A* regimen may be omitted if women receive >4 weeks of antenatal ZDV. The cited transmission risks reflect a range of antenatal ZDV durations, as well as studies both including and excluding the sdNVP and ZDV/3TC components. The Pediatric AIDS Clinical Trials Group 076 Study was conducted in a replacement-fed population. However, this study demonstrated MTCT risks at the upper bound of the published range, reducing concern for underestimation of early postpartum MTCT risk, and thus was used in the "highest risk" scenario.

**Supplemental Table 3. Derivation of unit costs for health services in Zimbabwe.**

|  | **Pre-conversion data from**  **WHO-CHOICE database** | | | | **Post-conversion to 2008 U.S. dollars** | |
| --- | --- | --- | --- | --- | --- | --- |
|  | *Local Currency Units* | | *International Dollars* | | *U.S. Dollars* | |
|  | Inpatient  bed-day | Outpatient visit | Inpatient bed-day | Outpatient visit | Inpatient bed-day | Outpatient visit |
|  | Tertiary facility | | | | | |
| Kenya | 665.73 | 221.68 | 34.99 | 11.65 | 12.33 | 4.10 |
| Malawi | 424.56 | 117.85 | 12.06 | 3.35 | 4.16 | 1.16 |
| Mozambique | 147.85 | 45.06 | 20.84 | 6.35 | 7.73 | 2.35 |
| Tanzania | 7690.75 | 2246.11 | 16.24 | 4.74 | 8.14 | 2.38 |
| Uganda | 9523.03 | 3114.3 | 31.48 | 10.3 | 6.48 | 2.12 |
| Zambia | 54,222.95 | 16,567.65 | 21.15 | 6.46 | 20.92 | 6.39 |
| **Average** |  |  |  |  | **9.96** | **3.08** |

**Supplemental Table 4. Intermediate (CEPAC model) results^a^**

| **I. Maternal outcomes (CEPAC adult model results)** | | | | | | |
| --- | --- | --- | --- | --- | --- | --- |
| **Maternal HIV status and HIV-related care at 6 weeks postpartum** | | **Life expectancy (undiscounted years, from delivery)** | | | **Lifetime per-person HIV-related costs (undiscounted, 2008 USD)** | |
| **ART-eligible ^b^** | |  | | |  | |
| On ART in pregnancy, continue lifelong | | 20.62 | | | **8,960** | |
| Not on ART in pregnancy, link to postnatal HIV care^c^ (and begin ART during BF), no sdNVP exposure | | 20.59 | | | **8,930** | |
| Not on ART in pregnancy, link to postnatal HIV care^c^ (and begin ART during BF), after sdNVP exposure | | 20.19 | | | **8,880** | |
| Not on ART in pregnancy, not in postnatal HIV care,^d^ no sdNVP exposure | | 11.42 | | | **4,120** | |
| Not on ART in pregnancy, not in postnatal HIV care,^d^ after sdNVP exposure | | 11.21 | | | **4,080** | |
| **Not ART-eligible ^b^** | |  | | |  | |
| On ART in pregnancy, continue lifelong | | 23.74 | | | **10,440** | |
| Not on ART in pregnancy, initiate ART at 6-week PP visit (for BF prophylaxis), continue lifelong, after sdNVP exposure | | 23.34 | | | **10,410** | |
| On ART in pregnancy and BF, interrupt at weaning | | 21.97 | | | **8,290** | |
| Not on ART in pregnancy, initiate ART at 6-week PP visit (for BF prophylaxis), interrupt at weaning, after sdNVP exposure, | | 21.32 | | | **8,220** | |
| Not on ART in pregnancy or BF, in postnatal HIV care, no sdNVP exposure | | 21.90 | | | **8,360** | |
| Not on ART in pregnancy or BF, in postnatal HIV care, after sdNVP exposure | | 21.55 | | | **8,330** | |
| Not in postnatal HIV care,^d^ no sdNVP exposure | | 14.84 | | | **4,470** | |
| Not in postnatal HIV care,^d^ after sdNVP exposure | | 14.64 | | | **4,440** | |
| **II. Pediatric outcomes (CEPAC infant model results)** | | | | | | |
| **Infant and maternal HIV status and HIV-related care**  **During breastfeeding** | **18-month HIV infection risk (%)** | | **Life expectancy (undiscounted years, from delivery)** | | | **Lifetime per-person**  **HIV-related costs (undiscounted, 2008 USD)** |
| **Infant HIV-uninfected at birth** | | | | | |  |
| Mother ART-eligible, on ART | 5.6 | | 45.60 | | | 374 |
| Mother ART-eligible, not in care/not on ART^c^ | 18.6 | | 41.86 | | | 621 |
| Mother not ART-eligible, on ART (*Options B/B+)* | 3.1 | | 46.29 | | | 328 |
| Mother not ART-eligible, infant on NVP (*Option A*) | 3.7 | | 46.10 | | | 496 |
| Mother not ART-eligible, no ARVs^c^ | 6.6 | | 45.31 | | | 393 |
| **Infant HIV-infected at birth** |  | | | | |  |
| Infant HIV diagnosis and ART available | n/a | | | 20 | | 2,180 |
| Infant HIV diagnosis and ART not available^c^ | n/a | | | 1.1 | | 272 |

**BF:** breastfeeding; **sdNVP:** single-dose nevirapine

a. CEPAC model outputs were intermediate results, used as inputs to the MTCT model.

b. ART eligibility was defined as CD4 ≤350/µL, or WHO stage 3-4 disease .

c. In the base-case analyses, women and infants were assumed to receive guideline-concordant antenatal care, and to link to postnatal care (defined as enrollment in HIV-related care by 6 weeks postpartum). Results are also shown for women and infants not in care during pregnancy and the postpartum period, to permit sensitivity analyses on the rates of PMTCT service uptake, loss to follow-up, and pediatric ART availability.

d. Women who do not link to postnatal HIV care were assumed to present to HIV care upon development of a severe opportunistic infection.

**Supplemental Table 5. Cost-effectiveness of WHO 2010 PMTCT guidelines in** Zimbabwe: sensitivity analyses

|  | **Costs**  **(discounted, 2008 USD) ^a^** | | | | **Life expectancy**  **(discounted, years from delivery)** | | | **Cost-effectiveness** |
| --- | --- | --- | --- | --- | --- | --- | --- | --- |
| **PMTCT regimen** | **ANC** | **Pediatric** | **Maternal** | **Total** | **Pediatric** | **Maternal** | **Total** | **ICER ($/YLS)** |
| **I. Base-case projections (from main manuscript, for comparison)** | | | | | | | | |
| **(100% PMTCT uptake, 100% retention in postnatal care, 100% pediatric ART availability)** | | | | | | | | |
| *Option B* | 134 | 240 | **5,260** | **5,630** | 23.59 | 14.74 | 38.32 |  |
| *Option A ^b^* | 118 | 310 | **5,280** | **5,710** | 23.19 | 14.70 | 37.89 | Dominated**^c^** |
| *sdNVP* | 92 | 360 | **5,300** | **5,760** | 22.45 | 14.53 | 36.97 | Dominated |
| *No antenatal ARVs* | 85 | 520 | **5,280** | **5,880** | 21.34 | 14.69 | 36.03 | Dominated |
| *Option B+* | 134 | 240 | **6,240** | **6,620** | 23.59 | 15.45 | 39.04 | **1,370** |
| **II. Access to care parameters** | | | | | | | | |
| **56% PMTCT uptake,^d^ 87% linkage to postnatal care** | | | | | | | | |
| *Option B* | 112 | 380 | **4,440** | **4,930** | 22.50 | 13.19 | 35.69 |  |
| *Option A* | 102 | 420 | **4,460** | **4,980** | 22.28 | 13.16 | 35.44 | Dominated |
| *sdNVP* | 85 | 450 | **4,460** | **5,000** | 21.85 | 13.07 | 34.92 | Dominated |
| *No antenatal ARVs* | 81 | 530 | **4,450** | **5,060** | 21.23 | 13.16 | 34.39 | Dominated |
| *Option B+* | 112 | 380 | **5,110** | **5,600** | 22.50 | 13.67 | 36.18 | **1,370** |
| **80% PMTCT uptake^c^** | | | | | | | | |
| *Option B* | 123 | 320 | **4,480** | **4,920** | 23.00 | 13.26 | 36.25 |  |
| *Option A* | 111 | 370 | **4,490** | **4,970** | 22.68 | 13.23 | 35.91 | Dominated |
| *sdNVP* | 90 | 410 | **4,510** | **5,010** | 22.22 | 13.10 | 35.32 | Dominated |
| *No antenatal ARVs* | 85 | 530 | **4,490** | **5,100** | 21.24 | 13.23 | 34.46 | Dominated |
| *Option B+* | 123 | 320 | **5,160** | **5,600** | 23.00 | 13.75 | 36.75 | **1,370** |
| **90% PMTCT uptake^c^** | | | | | | | | |
| *Option B* | 129 | 290 | **4,700** | **5,110** | 23.25 | 13.67 | 36.92 |  |
| *Option A* | 115 | 340 | **4,720** | **5,170** | 22.89 | 13.64 | 36.54 | Dominated |
| *sdNVP* | 91 | 390 | **4,730** | **5,210** | 22.25 | 13.50 | 35.75 | Dominated |
| *No antenatal ARVs* | 85 | 530 | **4,710** | **5,320** | 21.27 | 13.64 | 34.91 | Dominated |
| *Option B+* | 129 | 290 | **5,460** | **5,880** | 23.25 | 14.23 | 37.48 | **1,370** |
| **95% PMTCT uptake^c^** | | | | | | | | |
| *Option B* | 131 | 270 | **4,810** | **5,210** | 23.37 | 13.88 | 37.25 |  |
| *Option A* | 117 | 330 | **4,830** | **5,270** | 23.00 | 13.85 | 36.85 | Dominated |
| *sdNVP* | 91 | 380 | **4,840** | **5,310** | 22.32 | 13.70 | 36.02 | Dominated |
| *No antenatal ARVs* | 85 | 530 | **4,820** | **5,430** | 21.28 | 13.85 | 35.13 | Dominated |
| *Option B+* | 131 | 270 | **5,620** | **6,020** | 23.37 | 14.47 | 37.84 | **1,370** |
| **Increased maternal loss to follow-up after delivery (16% year 1, 6%/ year thereafter)** | | | | | | | | |
| *Option B* | 134 | 240 | **3,050** | **3,420** | 23.59 | 11.64 | 35.23 |  |
| *Option A* | 118 | 310 | **3,130** | **3,560** | 23.19 | 11.72 | 34.90 | Dominated |
| *sdNVP* | 92 | 360 | **3,170** | **3,620** | 22.45 | 11.61 | 34.06 | Dominated |
| *No antenatal ARVs* | 85 | 520 | **3,130** | **3,730** | 21.34 | 11.71 | 33.05 | Dominated |
| *Option B+* | 134 | 240 | **3,540** | **3,910** | 23.59 | 12.22 | 35.81 | **850** |
| **Reduced pediatric ART availability (36% of infected children; 2009 Zimbabwe estimate)** | | | | | | | | |
| *Option B* | 134 | 220 | **5,260** | **5,610** | 23.26 | 14.74 | 38.00 |  |
| *sdNVP* | 92 | 270 | **5,300** | **5,670** | 21.44 | 14.53 | 35.96 | Dominated |
| *Option A* | 118 | 270 | **5,280** | **5,670** | 22.71 | 14.70 | 37.41 | Dominated |
| *No antenatal ARVs* | 85 | 330 | **5,280** | **5,690** | 19.36 | 14.69 | 34.06 | Dominated |
| *Option B+* | 134 | 220 | **6,240** | **6,590** | 23.26 | 15.45 | 38.71 | **1,370** |
| **Current access to care (56% PMTCT uptake, 87% linkage to postnatal maternal care, increased maternal LTFU,**  **36% pediatric ART availability)** | | | | | | | | |
| *Option B* | 112 | 280 | **2,620** | **3,010** | 21.41 | 10.58 | 31.99 |  |
| *sdNVP* | 85 | 310 | **2,690** | **3,090** | 20.37 | 10.57 | 30.94 | Dominated |
| ***Option A*** | 102 | 320 | **2,680** | **3,090** | 21.10 | 10.62 | 31.72 | Dominated |
| ***No antenatal ARVs*** | 81 | 340 | **2,670** | **3,100** | 19.21 | 10.62 | 29.83 | Dominated |
| *Option B+* | 112 | 280 | **2,950** | **3,340** | 21.41 | 10.96 | 32.38 | **850** |
|  |  |  |  |  |  |  |  |  |

**Supplemental Table 5, continued.**

|  | **Costs**  **(discounted, 2008 USD) ^a^** | | | | | **Life expectancy**  **(discounted, years from delivery)** | | | **Cost-effectiveness** |
| --- | --- | --- | --- | --- | --- | --- | --- | --- | --- |
| **PMTCT regimen** | **ANC** | **Pediatric** | | **Maternal** | **Total** | **Pediatric** | **Maternal** | **Total** | **ICER ($/YLS)** |
| **II. Access to care parameters, continued** | | | | | | | | | |
| **CD4 availability in *Options A, B,* and *B+:* 25%** | | | | | | | | | |
| *Option B* | 127 | 240 | **5,260** | | **5,630** | 23.59 | 14.74 | 38.32 |  |
| *Option A* | 107 | 330 | **5,280** | | **5,720** | 22.97 | 14.70 | 37.67 | Dominated |
| *sdNVP* | 92 | 360 | **5,300** | | **5,760** | 22.45 | 14.53 | 36.97 | Dominated |
| *No antenatal ARVs* | 85 | 520 | **5,280** | | **5,880** | 21.34 | 14.69 | 36.03 | Dominated |
| *Option B+* | 127 | 240 | **6,240** | | **6,610** | 23.59 | 15.45 | 39.04 | **1,370** |
| **CD4 availability in *Options A, B,* and *B+:* 50%** | | | | | | | | | |
| *Option B* | 129 | 240 | **5,260** | | **5,630** | 23.59 | 14.74 | 38.32 |  |
| *Option A* | 111 | 330 | **5,280** | | **5,720** | 23.04 | 14.70 | 37.74 | Dominated |
| *sdNVP* | 92 | 360 | **5,300** | | **5,760** | 22.45 | 14.53 | 36.97 | Dominated |
| *No antenatal ARVs* | 85 | 520 | **5,280** | | **5,880** | 21.34 | 14.69 | 36.03 | Dominated |
| *Option B+* | 129 | 240 | **6,240** | | **6,610** | 23.59 | 15.45 | 39.04 | **1,370** |
| **CD4 availability in *Options A, B,* and *B+:* 75%** | | | | | | | | | |
| *Option B* | 131 | 240 | **5,260** | | **5,630** | 23.59 | 14.74 | 38.32 |  |
| *Option A* | 115 | 320 | **5,280** | | **5,720** | 23.12 | 14.70 | 37.81 | Dominated |
| *sdNVP* | 92 | 360 | **5,300** | | **5,760** | 22.45 | 14.53 | 36.97 | Dominated |
| *No antenatal ARVs* | 85 | 520 | **5,280** | | **5,880** | 21.34 | 14.69 | 36.03 | Dominated |
| *Option B+* | 131 | 240 | **6,240** | | **6,620** | 23.59 | 15.45 | 39.04 | **1,370** |
| **III. Clinical health parameters: Variation in life expectancy assumptions for infants**  **(base case: exposed/uninfected = 50 years, infected on ART = 20 years, ∆ = 30 years)** | | | | | | | | | |
| **High pediatric life expectancy assumptions (exposed/uninfected = 67 years, infected on ART = 25 years, ∆ = 42 years)** | | | | | | | | | |
| *Option B* | 134 | 260 | **5,260** | | **5,650** | 26.26 | 14.74 | 41.00 |  |
| *Option A* | 118 | 340 | **5,280** | | **5,740** | 25.83 | 14.70 | 40.53 | Dominated |
| *sdNVP* | 92 | 410 | **5,300** | | **5,800** | 25.06 | 14.53 | 39.59 | Dominated |
| *No antenatal ARVs* | 85 | 590 | **5,280** | | **5,950** | 23.93 | 14.69 | 38.62 | Dominated |
| *Option B+* | 134 | 260 | **6,240** | | **6,640** | 26.26 | 15.45 | 41.71 | **1,370** |
| **Low pediatric life expectancy assumptions (exposed/uninfected = 43 years, infected on ART = 10 years, ∆ = 33 years)** | | | | | | | | | |
| *Option B* | 134 | 200 | **5,260** | | **5,590** | 21.79 | 14.74 | 36.53 |  |
| *sdNVP* | 92 | 270 | **5,300** | | **5,660** | 20.27 | 14.53 | 34.79 | Dominated |
| *Option A* | 118 | 260 | **5,280** | | **5,660** | 21.32 | 14.70 | 36.02 | Dominated |
| *No antenatal ARVs* | 85 | 350 | **5,280** | | **5,710** | 18.66 | 14.69 | 33.35 | Dominated |
| *Option B+* | 134 | 200 | **6,240** | | **6,580** | 21.79 | 15.45 | 37.25 | **1,370** |
| **Largest difference in pediatric life expectancy assumptions between HIV-infected and HIV-uninfected infants (exposed/uninfected = 67 years, infected on ART = 10 years, ∆ = 57 years)** | | | | | | | | | |
| *Option B* | 134 | 210 | **5,260** | | **5,600** | 25.76 | 14.74 | 40.50 |  |
| *sdNVP* | 92 | 270 | **5,300** | | **5,670** | 23.81 | 14.53 | 38.34 | Dominated |
| *Option A* | 118 | 270 | **5,280** | | **5,670** | 25.71 | 14.70 | 39.87 | Dominated |
| *No antenatal ARVs* | 85 | 360 | **5,280** | | **5,720** | 21.74 | 14.69 | 36.43 | Dominated |
| *Option B+* | 134 | 210 | **6,240** | | **6,590** | 25.76 | 15.45 | 15.45 | **1,370** |
| **Smallest difference in pediatric life expectancy assumptions between HIV-infected and HIV-uninfected infants (exposed/uninfected = 43 years, infected on ART = 25 years, ∆ = 18 years)** | | | | | | | | | |
| *No antenatal ARVs* | 85 | 250 | **5,280** | | **5,620** | 20.85 | 14.69 | 35.55 |  |
| *sdNVP* | 92 | 400 | **5,300** | | **5,790** | 21.52 | 14.53 | 36.05 | Dominated |
| *Option B* | 134 | 550 | **5,260** | | **5,940** | 22.29 | 14.74 | 37.03 | 220 |
| *Option A* | 118 | 590 | **5,280** | | **6,000** | 21.98 | 14.70 | 36.68 | Dominated |
| *Option B+* | 134 | 550 | **6,240** | | **6,930** | 22.29 | 15.45 | 37.74 | **1,370** |

**Supplemental Table 5, continued**

| **PMTCT regimen** | **Costs**  **(2008 USD)** | | | | | | | | | **18m**  **HIV risk** | | **Life expectancy**  **(years from delivery)** | | | | | | **Cost-effectiveness** | |
| --- | --- | --- | --- | --- | --- | --- | --- | --- | --- | --- | --- | --- | --- | --- | --- | --- | --- | --- | --- |
|  | **ANC** | | **Pediatric** | | **Maternal** | | **Total** | | |  |  | **Pediatric** | | | **Maternal** | | **Total** | **ICER ($/YLS)** | |
| **IV. Clinical health parameters: Variation in MTCT risks associated with each modeled regimen** | | | | | | | | | | | | | | | | | | | |
| **"Best-case" MTCT risks** | | | | | | | | | | | | | | | | | | | |
| *Option B* | 134 | | 170 | | **5,260** | | | **5,560** | | 0.7% | | 24.08 | | 14.74 | | | 38.81 |  | |
| *sdNVP* | 92 | | 240 | | **5,300** | | | **5,630** | | 5.5% | | 23.31 | | 14.53 | | | 37.83 | Dominated | |
| *Option A* | 118 | | 250 | | **5,280** | | | **5,650** | | 3.1% | | 23.62 | | 14.70 | | | 38.32 | Dominated | |
| *No antenatal ARVs* | 85 | | 390 | | **5,280** | | | **5,750** | | 16.0% | | 22.22 | | 14.69 | | | 36.91 | Dominated | |
| *Option B+* | 134 | | 170 | | **6,240** | | | **6,550** | | 0.7% | | 24.08 | | 15.45 | | | 39.53 | **1,370** | |
| **"Worst-case" MTCT risks** | | | | | | | | | | | | | | | | | | | |
| *Option B* | 134 | | 330 | | **5,260** | | | **5,720** | | 11.5% | | 23.01 | | 14.74 | | | 37.75 |  | |
| *Option A* | 118 | | 350 | | **5,280** | | | **5,750** | | 10.1% | | 22.94 | | 14.70 | | | 37.64 | Dominated | |
| *sdNVP* | 92 | | 480 | | **5,300** | | | **5,870** | | 21.9% | | 21.68 | | 14.53 | | | 36.21 | Dominated | |
| *No antenatal ARVs* | 85 | | 610 | | **5,280** | | | **5,980** | | 31.4% | | 20.68 | | 14.69 | | | 35.38 | Dominated | |
| *Option B+* | 134 | | 330 | | **6,240** | | | **6,700** | | 11.5% | | 23.01 | | 15.45 | | | 38.47 | **1,370** | |
| **Equal antenatal and postnatal MTCT risks for *Options A* and *B* with maternal CD4 >350/µL** | | | | | | | | | | | | | | | | | | | |
| *Option B* | 134 | | 240 | | **5,260** | | | **5,630** | | 5.7% | | 23.59 | | 14.74 | | | 38.32 |  | |
| *Option A* | 118 | | 290 | | **5,280** | | | **5,690** | | 5.7% | | 23.38 | | 14.70 | | | 38.08 | Dominated | |
| *sdNVP* | 92 | | 360 | | **5,300** | | | **5,750** | | 14.2% | | 22.45 | | 14.53 | | | 36.97 | Dominated | |
| *No antenatal ARVs* | 85 | | 520 | | **5,280** | | | **5,890** | | 24.8% | | 21.34 | | 14.69 | | | 36.03 | Dominated | |
| *Option B+* | 134 | | 240 | | **6,240** | | | **6,610** | | 5.7% | | 23.59 | | 15.45 | | | 39.04 | **1,370** | |
| **V. Clinical health parameters: Variation in maternal healthcare parameters** | | | | | | | | | | | | | | | | | | | |
|  | | **Costs**  **(discounted, 2008 USD) ^a^** | | | | | | | | | **Life expectancy**  **(discounted, years from delivery)** | | | | | | | | **Cost-effectiveness** |
| **PMTCT regimen** | | **ANC** | | **Pediatric** | | **Maternal** | | | **Total** | | **Pediatric** | | **Maternal** | | | **Total** | | | **ICER ($/YLS)** |
| **Drug resistance due to ART-interruption: reduced efficacy (90%) of NNRTI-based ART when restarted after ART interruption (*Option B*) (base-case: 100%)** | | | | | | | | | | | | | | | | | | | |
| *Option B* | | 134 | | 240 | | **5,330** | | | **5,710** | | 23.59 | | 14.58 | | | 38.16 | | |  |
| *Option A* | | 118 | | 310 | | **5,280** | | | **5,710** | | 23.19 | | 14.70 | | | 37.89 | | | Dominated |
| *sdNVP* | | 92 | | 360 | | **5,300** | | | **5,760** | | 22.45 | | 14.53 | | | 36.97 | | | Dominated |
| *No antenatal ARVs* | | 85 | | 520 | | **5,280** | | | **5,880** | | 21.34 | | 14.69 | | | 36.03 | | | Dominated |
| *Option B+* | | 134 | | 240 | | **6,290** | | | **6,670** | | 23.59 | | 15.45 | | | 39.04 | | | 1,100 |
| **"Treatment fatigue:" monthly risk of virologic failure after 6 months on 1st-line NNRTI-based ART for women starting ART with CD4>350/µL (*Options B/B+)* (base-case: 1.59%)** | | | | | | | | | | | | | | | | | | | |
| **Monthly risk = 1.99% (1.25x base-case risk)** | | | | | | | | | | | | | | | | | | | |
| *Option B* | | 134 | | 240 | | **5,310** | | | **5,680** | | 23.59 | | 14.39 | | | 37.98 | | |  |
| *Option A* | | 118 | | 310 | | **5,280** | | | **5,710** | | 23.19 | | 14.70 | | | 37.89 | | | Dominated |
| *sdNVP* | | 92 | | 360 | | **5,300** | | | **5,760** | | 22.45 | | 14.53 | | | 36.97 | | | Dominated |
| *No antenatal ARVs* | | 85 | | 520 | | **5,280** | | | **5,880** | | 21.34 | | 14.69 | | | 36.03 | | | Dominated |
| *Option B+* | | 134 | | 240 | | **6,290** | | | **6,670** | | 23.59 | | 15.23 | | | 38.82 | | | **1,170** |
| **Monthly risk = 2.19% (1.375x base-case risk)** | | | | | | | | | | | | | | | | | | | |
| *Option B* | | 134 | | 240 | | **5,320** | | | **5,700** | | 23.59 | | 14.30 | | | 37.89 | | |  |
| *Option A* | | 118 | | 310 | | **5,280** | | | **5,710** | | 23.19 | | 14.70 | | | 37.89 | | | Dominated ^e^ |
| *sdNVP* | | 92 | | 360 | | **5,300** | | | **5,760** | | 22.45 | | 14.53 | | | 36.97 | | | Dominated |
| *No antenatal ARVs* | | 85 | | 520 | | **5,280** | | | **5,880** | | 21.34 | | 14.69 | | | 36.03 | | | Dominated |
| *Option B+* | | 134 | | 240 | | **6,310** | | | **6,690** | | 23.59 | | 15.16 | | | 38.74 | | | **1,160** |
| **Monthly risk = 2.39% (1.5x base-case risk)** | | | | | | | | | | | | | | | | | | | |
| *Option B* | | 134 | | 240 | | **5,330** | | | **5,700** | | 23.59 | | 14.23 | | | 37.82 | | |  |
| *Option A* | | 118 | | 310 | | **5,280** | | | **5,710** | | 23.19 | | 14.70 | | | 37.89 | | | **190** |
| *sdNVP* | | 92 | | 360 | | **5,300** | | | **5,760** | | 22.45 | | 14.53 | | | 36.97 | | | Dominated |
| *No antenatal ARVs* | | 85 | | 520 | | **5,280** | | | **5,880** | | 21.34 | | 14.69 | | | 36.03 | | | Dominated |
| *Option B+* | | 134 | | 240 | | **6,330** | | | **6,700** | | 23.59 | | 15.08 | | | 38.67 | | | 1,260 |

**Supplemental Table 5, continued**

|  | **Costs**  **(discounted, 2008 USD) ^a^** | | | | **Life expectancy**  **(discounted, years from delivery)** | | | **Cost-effectiveness** |
| --- | --- | --- | --- | --- | --- | --- | --- | --- |
| **PMTCT regimen** | **ANC** | **Pediatric** | **Maternal** | **Total** | **Pediatric** | **Maternal** | **Total** | **ICER ($/YLS)** |
| **V. Clinical health parameters: Variation in maternal healthcare parameters, continued** | | | | | | | | |
| **"Treatment fatigue:" monthly risk of virologic failure after 6 months on 1st-line NNRTI-based ART for women starting ART with CD4>350/µL (*Options B/B+)* (base-case: 1.59%), continued** | | | | | | | | |
| **Monthly risk = 3.18% (2x base-case risk)** | | | | | | | | |
| ***Option A*** | **118** | **310** | **5,280** | **5,710** | **23.19** | **14.70** | **37.89** |  |
| ***Option B*** | **134** | **240** | **5,350** | **5,730** | **23.59** | **13.98** | **37.57** | Dominated |
| *sdNVP* | 92 | 360 | **5,300** | **5,760** | 22.45 | 14.53 | 36.97 | Dominated |
| *No antenatal ARVs* | 85 | 520 | **5,280** | **5,880** | 21.34 | 14.69 | 36.03 | Dominated |
| *Option B+* | 134 | 240 | **6,370** | **6,750** | 23.59 | 14.87 | 38.45 | **1,820** |
| **Monthly risk = 4.09% (2.5x base-case risk)** | | | | | | | | |
| *Option A* | 118 | 310 | **5,280** | **5,710** | 23.19 | 14.70 | 37.89 |  |
| *Option B* | 134 | 240 | **5,370** | **5,750** | 23.59 | 13.81 | 37.40 | Dominated |
| *sdNVP* | 92 | 360 | **5,300** | **5,760** | 22.45 | 14.53 | 36.97 | Dominated |
| *No antenatal ARVs* | 85 | 520 | **5,280** | **5,880** | 21.34 | 14.69 | 36.03 | Dominated |
| *Option B+* | 134 | 240 | **6,410** | **6,780** | 23.59 | 14.69 | 38.28 | **2,710** |
| **Monthly risk = 5.00%** | | | | | | | | |
| *Option A* | 118 | 310 | **5,280** | **5,710** | 23.19 | 14.70 | 37.89 |  |
| ***sdNVP*** | 92 | 360 | **5,300** | **5,760** | 22.45 | 14.53 | 36.97 | Dominated |
| ***Option B*** | 134 | 240 | **5,390** | **5,760** | 23.59 | 13.71 | 37.29 | Dominated |
| *No antenatal ARVs* | 85 | 520 | **5,280** | **5,880** | 21.34 | 14.69 | 36.03 | Dominated |
| *Option B+* | 134 | 240 | **6,430** | **6,810** | 23.59 | 14.59 | 38.17 | **3,820** |
| **"Treatment fatigue": reduced efficacy (60%) of 2nd-line PI based ART for women starting ART with CD4>350/µL (*Options B/B+) (*base-case: 72%)** | | | | | | | | |
| *Option B* | 134 | 240 | **5,120** | **5,500** | 23.59 | 14.50 | 38.08 |  |
| *Option A* | 118 | 310 | **5,280** | **5,710** | 23.19 | 14.70 | 37.89 | Dominated |
| *sdNVP* | 92 | 360 | **5,300** | **5,760** | 22.45 | 14.53 | 36.97 | Dominated |
| *No antenatal ARVs* | 85 | 520 | **5,280** | **5,880** | 21.34 | 14.69 | 36.03 | Dominated |
| *Option B+* | 134 | 240 | **6,090** | **6,470** | 23.59 | 15.19 | 38.77 | **1,400** |
| **VI. Resource utilization parameters** | | | | | | | | |
| **South Africa healthcare costs ^f^** | | | | | | | | |
| *Option B* | 134 | 2,230 | **11,680** | **14,040** | 23.59 | 14.74 | 38.33 |  |
| *Option A* | 118 | 2,400 | **11,740** | **14,260** | 23.19 | 14.70 | 37.89 | Dominated |
| *sdNVP* | 92 | 2,880 | **11,760** | **14,730** | 22.45 | 14.53 | 36.97 | Dominated |
| *Option B+* | 134 | 2,230 | **12,710** | **15,070** | 23.59 | 15.47 | 39.05 | 1,410 |
| *No antenatal ARVs* | 85 | 3,700 | **11,730** | **15,520** | 21.34 | 14.69 | 36.04 | Dominated |
| **6 months antenatal drug regimens (base-case: 2 months) ^g^** | | | | | | | | |
| *Option B* | 202 | 240 | **5,260** | **5,700** | 23.59 | 14.74 | 38.32 |  |
| *Option A* | 163 | 310 | **5,280** | **5,760** | 23.19 | 14.70 | 37.89 | Dominated |
| *sdNVP* | 100 | 360 | **5,300** | **5,770** | 22.45 | 14.53 | 36.97 | Dominated |
| *No antenatal ARVs* | 85 | 520 | **5,280** | **5,880** | 21.34 | 14.69 | 36.03 | Dominated |
| *Option B+* | 202 | 240 | **6,240** | **6,690** | 23.59 | 15.45 | 39.04 | **1,370** |
| **Pediatric ART costs: lifelong LPV/r-based ART for infected infants after NVP exposure (*sdNVP, Option A*)^h^** | | | | | | | | |
| *Option B* | 134 | 240 | **5,260** | **5,630** | 23.59 | 14.74 | 38.32 |  |
| *Option A* | 118 | 330 | **5,280** | **5,730** | 23.19 | 14.70 | 37.89 | Dominated |
| *No antenatal ARVs* | 85 | 520 | **5,280** | **5,880** | 21.34 | 14.69 | 36.03 | Dominated |
| *sdNVP* | 92 | 530 | **5,300** | **5,920** | 22.45 | 14.53 | 36.97 | Dominated |
| *Option B+* | 134 | 240 | **6,240** | **6,620** | 23.59 | 15.45 | 39.04 | **1,370** |

**ANC**: antenatal care; **ICER**: incremental cost-effectiveness ratio; **YLS**: year of life saved; **ARVs**: antiretroviral drugs; **ART**:

three-drug antiretroviral therapy (for treatment of HIV infection), **LTFU**: loss to follow-up, **LPV/r:** lopinavir/ritonavir; **NVP**: nevirapine.

**Supplemental Table 5, continued (footnotes):**

a. All costs (except ANC costs, with time horizon of 2-6 months) are discounted at 3% annually for cost-effectiveness analyses.

b. The negligible difference in projected life expectancy between *no ARVs* and *Option A* is due to two factors: 1) the assumption that a short course of zidovudine during pregnancy only minimally impacts disease progression , and 2) that all women who require ART will begin it postpartum, attenuating the differences between strategies with only antenatal and intrapartum components.

c. A “dominated” strategy is one that is more expensive, but less effective, than an alternative strategy.

d. PMTCT uptake is defined as the proportion of HIV-infected, pregnant women receiving medications for PMTCT by the time of delivery (see Supplemental Table 2, Section I).

e. At the 2.19% value for late risk of virologic failure (with greater costs but nearly equivalent LE for *Option A*, compared to *Option B*), demonstrates the threshold at which *Option B* ceases to dominate *Option A* by “strong” dominance; that is, above this value, *Option B* is no longer more expensive and less effective. At this value, *Option B* dominates *Option A* by “weak” or “extended” dominance. This means that while more expensive and (very slightly) more effective than *Option B*, *Option A* is not enough more effective to warrant its greater cost, and represents an inefficient use of healthcare resources.

f. In analyses using South Africa healthcare costs, ART costs are held equal to the base case (as they derive from international donor price lists applicable to both Zimbabwe and South Africa). In addition, ANC costs were not changed in the “South Africa healthcare costs” scenario, as these inputs were derived in the base case in USD from a variety of Southern African sources. The costs of postnatal clinical care, however, are greater than in the base case.

g. A scenario is modeled in which antenatal ARVs are received for six months (base-case: 2 months); this is modeled as increase in costs, without change in efficacy, due to the wide variation of antenatal ARV duration in the PMTCT trials informing the MTCT risk estimates.

h. In this sensitivity analysis, we applied the costs of lifelong LPV/r-based ART to HIV-infected infants exposed to sdNVP for PMTCT. This included all infants in the *sdNVP* strategy, and 25% of the infants of non-ART-eligible women in *Option A* (based on a median gestational age at ARV initiation of 30 weeks (IQR 24-36 weeks), 25% of women with CD4 >350/µL would receive <4 weeks of antenatal ZDV, and thus be offered sdNVP in labor).

**Supplemental Table 6: Impact of hypothetical "implementation cost" for three-drug ARV regimens during pregnancy**

|  | **Lifetime projections, per mother-infant pair (discounted)** | | | |
| --- | --- | --- | --- | --- |
| **Regimen** | **Combined LE** | **Total cost (2008 USD)** | **ICER ($/YLS)** | **Policy conclusions** |
| **Cost difference, three-drug ARVs vs. ZDV = $125 ^a^** | | | | |
| ***Option B*** | **38.32** | **5,740** |  |  |
| ***Option A*** | **37.89** | **5,750** | **Dominated ^b^** |  |
| ***sdNVP*** | **36.97** | **5,770** | **Dominated** | **Policy conclusions unchanged from base case** |
| ***No ARVs*** | **36.03** | **5,880** | **Dominated** |  |
| ***Option B+*** | **39.04** | **6,720** | **1,370** |  |
| **Cost difference, three-drug ARVs vs. ZDV = $150** | | | | |
| *Option A* | 37.89 | **5,760** |  | *Option A* still dominates *sdNVP*  *Option B* no longer dominates *Option A*, but is very CE compared to *Option A* **($2/YLS**) **^c^**  *Option A, sdNVP,* and *Option B* still dominate *no antenatal ARVs*  *Option B+* vs. *Option B* unchanged |
| *Option B* | 38.32 | **5,760** | **2** |
| *sdNVP* | 36.97 | **5,770** | Dominated |
| *No ARVs* | 36.03 | **5,880** | Dominated |
| *Option B+* | 39.04 | **6,750** | **1,370** |
| **Cost difference, three-drug ARVs vs. ZDV = $175** | | | | |
| *Option A* | 37.89 | **5,770** |  | *Option A* still dominates *sdNVP*  ***Option B remains* very CE compared to *Option A* ($40/YLS)**  *Option A, sdNVP,* and *Option B* still dominate *no antenatal ARVs*  *Option B+* vs. *Option B* unchanged |
| *sdNVP* | 36.97 | **5,780** | Dominated |
| *Option B* | 38.32 | **5,780** | **40** |
| *No ARVs* | 36.03 | **5,880** | Dominated |
| *Option B+* | 39.04 | **6,770** | **1,370** |
| **Cost difference, three-drug ARVs vs. ZDV = $200** | | | | |
| ***Option A*** | 37.89 | **5,780** |  | *Option A* is on the threshold to no longer dominate sdNVP (nearly equivalent cost, but greater LE).  *Option B* remains very CE compared to *Option A* **($80/YLS)**  *Option A, sdNVP,* and *Option B* still dominate *no antenatal ARVs*  *Option B+* vs. *Option B* unchanged |
| ***sdNVP*** | 36.97 | **5,780** | **Dominated** |
| *Option B* | 38.32 | **5,810** | **80** |
| *No ARVs* | 36.03 | **5,880** | Dominated |
| *Option B+* | 39.04 | **6,790** | **1,370** |
| **Cost difference, three-drug ARVs vs. ZDV = $250** | | | | |
| *sdNVP* | 36.97 | **5,790** |  | *Option A* no longer dominates *sdNVP*, but is very cost-effective compared to *sdNVP* ($10/YLS)  *Option B* remains very CE compared to *Option A* **($150/YLS)**  *Option A, sdNVP,* and *Option B* still dominate *no antenatal ARVs*  *Option B+* vs. *Option B* unchanged |
| *Option A* | 37.89 | **5,790** | 10 |
| *Option B* | 38.32 | **5,860** | **150** |
| *No ARVs* | 36.03 | **5,880** | Dominated |
| *Option B+* | 39.04 | **6,840** | **1,370** |
| **Cost difference, three-drug ARVs vs. ZDV = $275** | | | | |
| *sdNVP* | 36.97 | **5,790** |  | *Option A* is very cost-effective compared to *sdNVP* ($20/YLS)  *Option B* no longer dominates *no antenatal ARVs*, but *Option A* and *sdNVP* still dominate *no antenatal ARVs*  *Option B* remains very CE compared to *Option A* ($180/YLS)  *Option B+* vs. *Option B* unchanged |
| *Option A* | 37.89 | **5,800** | 20 |
| *No ARVs* | 36.03 | **5,880** | Dominated |
| *Option B* | 38.32 | **5,880** | 180 |
| *Option B+* | 39.04 | **6,870** | **1,370** |
| **Cost difference, three-drug ARVs vs. ZDV = $300** | | | | |
| *sdNVP* | 36.97 | **5,790** |  | *Option A* is very cost-effective compared to *sdNVP* **($20/YLS)**  *Option A* and *sdNVP* still dominate *no antenatal ARVs*  *Option B* remains very CE compared to *Option A* ($220/YLS)  *Option B+* vs. *Option B* unchanged |
| *Option A* | 37.89 | **5,810** | **20** |
| *No ARVs* | 36.03 | **5,880** | Dominated |
| *Option B* | 38.32 | **5,910** | 220 |
| *Option B+* | 39.04 | **6,890** | **1,370** |

**Supplemental Table 6, continued**

|  | **Lifetime projections, per mother-infant pair (discounted)** | | | |
| --- | --- | --- | --- | --- |
| **Regimen** | **Combined LE** | **Total cost (2008 USD)** | **ICER ($/YLS)** | **Policy conclusions** |
| **Cost difference, three-drug ARVs vs. ZDV = $400** | | | | |
| *sdNVP* | 36.97 | **5,810** |  | *Option A* is very cost-effective compared to *sdNVP* ($50/YLS)  *Option A* and *sdNVP* still dominate *no antenatal ARVs*  *Option B* remains very CE compared to *Option A* **($370YLS)**  *Option B+* vs. *Option B* unchanged |
| *Option A* | 37.89 | **5,850** | 50 |
| *No ARVs* | 36.03 | **5,880** | Dominated |
| *Option B* | 38.32 | **6,010** | **370** |
| *Option B+* | 39.04 | **6,990** | **1,370** |
| **Cost difference, three-drug ARVs vs. ZDV = $500** | | | | |
| *sdNVP* | 36.97 | **5,820** |  | *sdNVP* still dominates *no antenatal ARVs*  *Option A* no longer dominates *no antenatal ARVs*, but *Option A* is very CE compared to *sdNVP* **($70/YLS)**  *Option B* is now CE (not very CE) compared to  *Option A* ($510/YLS)  *Option B+* vs. *Option B* unchanged |
| *No ARVs* | 36.03 | **5,880** | Dominated |
| *Option A* | 37.89 | **5,880** | **70** |
| *Option B* | 38.32 | **6,110** | 510 |
| *Option B+* | 39.04 | **7,090** | **1,370** |

**ANC**: antenatal care; **ICER**: incremental cost-effectiveness ratio; **YLS**: year of life saved; **ARVs**: antiretroviral drugs; ZDV: zidovudine; **sdNVP**: single-dose nevirapine.

**a.** Cost difference includes all costs required to provide a three-drug ARV regimen in place of ZDV monotherapy, per patient receiving ARVs. The base case difference ($25) includes one full blood count and the additional cost of TDF/FTC/NVP (CD4 >350/µL) or TDF/FTC/EFV (CD4 ≤350/µL) compared to ZDV. If additional costs are required to provide three-drug regimens, for example to train healthcare personnel, scale up laboratory services, ensure stable drug supply, etc, these costs would need to equal at least $200/patient to change the comparison between *sdNVP* and *no antenatal ARVs*, and at least $275 to change the comparison between *Options A* and *B*.

**b.** Dominance: "strongly dominated" refers to an intervention that is more expensive and less effective than its alternative. "Weakly dominated," or dominated via "extended dominance," refers to an intervention that is more expensive and more effective than its alternative, but not sufficiently more effective to warrant its greater cost.

**c**. Based on WHO-recommended thresholds: “very cost-effective” = <1X Zimbabwe *per-capita* GDP ($400)/YLS; “cost-effective” = <3x GDP ($1,200)/YLS.

**Supplemental Table 7. Cumulative total costs over the first five years after delivery (undiscounted; input data for Manuscript Figure 2)**

|  | **Costs (undiscounted, 2008 USD)** | | | |
| --- | --- | --- | --- | --- |
| **Regimen** | **ANC** | **Pediatric ^a^** | **Maternal ^b^** | **Total ^c^** |
| **Year 1** |  |  |  |  |
| *No antenatal ARVs* | 85 | 42 | **136** | **263** |
| *sdNVP* | 92 | 33 | **138** | **263** |
| *Option A* | 118 | 60 | **138** | **316** |
| *Option B* | 134 | 28 | **238** | **400** |
| *Option B+* | 134 | 28 | **238** | **400** |
|  |  |  |  |  |
| **Year 2** |  |  |  |  |
| *No antenatal ARVs* | 85 | 83 | **257** | **425** |
| *sdNVP* | 92 | 67 | **264** | **423** |
| *Option A* | 118 | 105 | **259** | **482** |
| *Option B* | 134 | 56 | **383** | **573** |
| *Option B+* | 134 | 56 | **467** | **657** |
| **Year 3** |  |  |  |  |
| *No antenatal ARVs* | 85 | 123 | **404** | **612** |
| *sdNVP* | 92 | 101 | **418** | **610** |
| *Option A* | 118 | 134 | **406** | **658** |
| *Option B* | 134 | 84 | **499** | **717** |
| *Option B+* | 134 | 84 | **697** | **915** |
|  |  |  |  |  |
| **Year 4** |  |  |  |  |
| *No antenatal ARVs* | 85 | 163 | **595** | **844** |
| *sdNVP* | 92 | 134 | **620** | **845** |
| *Option A* | 118 | 162 | **598** | **878** |
| *Option B* | 134 | 112 | **629** | **875** |
| *Option B+* | 134 | 112 | **930** | **1,175** |
|  |  |  |  |  |
| **Year 5** |  |  |  |  |
| *No antenatal ARVs* | 85 | 200 | **816** | **1,101** |
| *sdNVP* | 92 | 164 | **854** | **1,110** |
| *Option A* | 118 | 188 | **818** | **1,125** |
| *Option B* | 134 | 137 | **803** | **1,074** |
| *Option B+* | 134 | 137 | **1,184** | **1,455** |

**a. Pediatric healthcare costs** include the costs of daily infant NVP in the Option A regimen. Costs of terminal pediatric care are excluded from short-term analyses due to model structure; if they were included, it is likely that effective PMTCT interventions would become cost-saving sooner after delivery.

**b. Maternal healthcare costs** include the costs of maternal three-drug ARVs in the *Option B* and *B+* regimens.

**c. Total costs for each year** are cumulative sums of antenatal care costs, pediatric healthcare costs, and maternal HIV-related healthcare costs.

**Supplemental Table 8. Comparison of UNAIDS/Spectrum and CEPAC estimates of MTCT for specific medication regimens.**

| IU/IP transmission | | PP transmission | |
| --- | --- | --- | --- |
| UNAIDS estimates | CEPAC estimates | UNAIDS estimates | CEPAC estimates |
| No ARVs | | | |
| CD4≤200/µL: 37%  Range: 22-54%  CD4 200-350/µL: 27%  Range: 13.1-32.6% | CD4≤350: 27.3%  Range: 19.9-32.2% | CD4 ≤350/µL: 1.57%/m  Range: 0.8-2.5%/m | CD4 ≤350/µL: 1.23%/m  Range: 0.5-2.3%/m |
| CD4>350/µL: 15%  Range: 9.7-20.2% | CD4 >350/µL: 17.4%  Range: 12.7-20.1% | CD4 >350/µL: 0.51%/m  Range: 0.1-0.96%/m | CD4 >350/µL: 0.39%/m  Range: 0.15-0.73%/m |
| sdNVP | | | |
| CD4 not specified: 12%  Range: 9.4-12.1% | CD4≤350/µL: 17.6%  Range: 8.1-26.4%  CD4>350/µL: 7.2%  Range: 3.3-10.9% | Assume = no ARVs | Assume = no ARVs |
| Zidovudine in pregnancy (Option A) | | Infant NVP in breastfeeding (Option A) | |
| CD4 200-350/µL: 4%  Range: 2.3-5.3%  CD4>350/µL: 2%  Range: 1.6-3.3% | CD4 ≤350/µL: 13.6%  Range: 9.1-15.7%  CD4 >350/µL: 3.6%  Range: 2.4-4.2% | CD4>350/µL: 0.2%/m  Range: 0.12-0.31%/m | CD4>350/µL: 0.22%/m  Range: 0.12-0.31%/m |
| ART in pregnancy (Option A/B) | | | |
| CD4 ≤350/µL: 2%  Range: 0.6-3.7% | CD4 ≤350/µL: 3.3%  Range: 1.1-4.1% | CD4 ≤350/µL: 0.2%/m  Range: 0-0.42% | CD4 ≤350/µL: 0.33%/m  Range: 0-0.53%/m |
| CD4>350/µL: 2%  Range: 0.9-2.9% | CD4 >350/µL: 1.0%  Range: 0.4-2.8% | CD4>350/µL: 0.2%/m  Range: 0.063-0.53% | CD4 >350/µL: 0.19%/m  Range: 0-0.53%/m |
| IU/IP: intrauterine/intrapartum transmission (by 4-6 weeks of age); sdNVP: single-dose nevirapine; EBF/MBF: exclusive/mixed breastfeeding | | | |

Notes. Key differences between the UNAIDS and CEPAC analyses included:

1. Calculation of postnatal transmission risks using conditional probabilities, conversion to monthly rates, and conversion back to monthly probabilities . The UNAIDS method instead divided difference in cumulative probability by time at risk. As a result, the CEPAC and UNAIDS estimates differ slightly, even when derived from the same data.

2. For intrauterine/intrapartum MTCT risk with *no ARVs*: a) use of a meta-analysis , which reduced the weight given by the UNAIDS team to a small West African cohort with very high MTCT risk (54%, ); and b) exclusion of postnatal transmission included in the UNAIDS estimates . These differences led to lower risks in the CEPAC analysis, rendering our analysis conservative with regard to the benefit of any PMTCT regimen.

3. For intrauterine/intrapartum MTCT risks among women with CD4 ≤350/µL receiving antenatal AZT (a component of *Option A*): use of data from women with CD4 of 0-350/µL , in comparison to data used by UNAIDS from women with CD4 of 200-350/µL. This difference led to higher risks in the CEPAC analysis; because it was applied in the model only when CD4 assays were not available, this permits the CEPAC model to project the impact of "real-world" implementation of *Option A*.

4. For the *Option A* regimens (antenatal AZT, neonatal NVP) among women with CD4 >350/µL: use of empiric data on intrauterine/intrapartum risk from women eligible for ART in the Ditrame Plus study , and use of a median value for postnatal risk from published studies in which infant medications continued throughout the observed period of breastmilk exposure . The UNAIDS group assigned risks based on expert consensus, and these risks were assumed to be equal for the *Options* *A* and *B* regimens. In sensitivity analyses in which we assumed equal risks for *Options A* and *B,* policy conclusions were unchanged (Supplemental Table 5, above).

5. When empiric data were not stratified by maternal CD4 ≤350/µL vs. >350/µL, CEPAC analyses applied published relative risks to assign higher MTCT risks to women with more advanced HIV disease; the UNAIDS team assigned equal risks regardless of maternal CD4.

**Supplemental Figure 1. PMTCT "cascade;" schematic representation of the MTCT model (adapted with permission from**

**Ciaranello *et al, PLoS ONE*, 2011; 6(6)).**


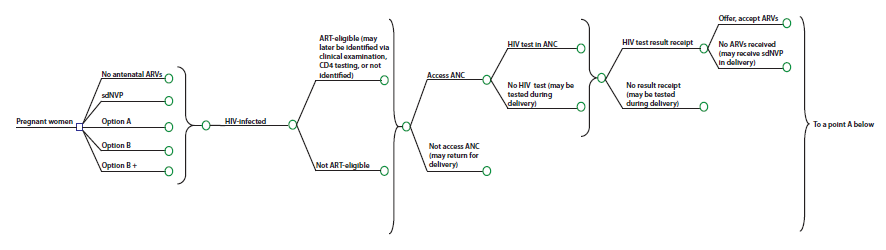


**LEGEND:** The MTCT model is a decision tree, coded in TreeAgePro software. Pregnant women enter the model at conception. The five modeled PMTCT strategies are shown at the decision node, indicated by a square. Circles indicate chance nodes, at which events occur based on probabilities derived from published literature. Triangles indicate terminal nodes, representing the clinical outcome of any single pathway through the model. Brackets reflect that the subsequent events emerging to the right of the bracket may follow any of the prior chance nodes included to the left of the bracket. At each chance node, the probabilities of all subsequent modeled events may depend on the PMTCT strategy being simulated and on the prior events leading to that node.

For each modeled PMTCT strategy, the series of events shown in the Figure may occur. For example, HIV-infected women may be ART-eligible (CD4≤350/µL or WHO Stage 3-4 disease) or non-ART-eligible; ART-eligibility may be identified by CD4 testing, identified by clinical evaluation, or not identified. All women may access ANC, undergo HIV testing in ANC, and receive HIV test results, or may fail to access these steps in the cascade.

If identified as HIV-infected, women may be offered ARVs for PMTCT according to the PMTCT strategy being simulated, as well as ART if identified as ART-eligible (not shown). In the base case, women were assumed to receive all ANC services; service uptake was varied in sensitivity analyses. Probabilities for surviving pregnancy depend on receipt of ART; if maternal death occurs, infant death also occurs. Women who survive pregnancy may deliver at a healthcare facility or at home; if they deliver in a healthcare facility, they may access HIV testing (if previous status was unknown or negative), and if identified as HIV-infected at that time, may receive sdNVP in labor. All women surviving pregnancy then experience probabilities of live birth and HIV infection in their infants, depending on PMTCT regimen received. Finally, women may link or fail to link to postnatal HIV-related care for themselves.

At the end (far right) of any given path through the model, there are two sets of outcomes: infant outcomes and maternal outcomes. Infant outcomes include HIV infection status (infected or uninfected at birth, shown), risk of postnatal HIV infection if uninfected at birth, life expectancy, and per-person healthcare costs. Maternal outcomes include life expectancy and per-person HIV-related healthcare costs. These outcomes are derived from the CEPAC adult and infant models, through specific simulations of each possible scenario described at the end of the pathways shown in the MTCT model. As an example of infant outcomes from the CEPAC infant model, an HIV-uninfected infant with an ART-eligible mother who is in postnatal care (and thus on ART) would face monthly risks of HIV infection based on receipt of maternal ART during breastfeeding (details shown in Supplemental Figure 2b, below), leading to a LE and lifetime cost projection for the infant. As an example of maternal outcomes from the CEPAC adult model, this infant’s mother would face CD4-dependent monthly risks of OI’s, ART failure or toxicity, and AIDS-related and AIDS-unrelated death, leading to a LE and lifetime cost projection for herself. These CEPAC model outputs are then used as “payoffs” (outcomes) in the MTCT model, according to conventional methods for evaluation of a decision tree. The average value assigned to any modeled PMTCT strategy in the MTCT model is, in essence, a weighted average of the value of these outcomes at the end of each pathway (weighted by the probabilities of reaching each possible path endpoint).

**Supplemental Figure 2. Schematic representations of the CEPAC adult and infant models (adapted with permission from Ciaranello *et al, PLoS ONE*, 2011; 6(6)).**

**LEGEND**. **Schematic representations of the adult and infant CEPAC model structures.**

Women enter the adult model (Supplemental Figure 2a) after delivery; for this analysis, all modeled women enter with chronic HIV infection. They then face monthly risks of clinical events including opportunistic infections, medication toxicities, and death; these risks are stratified by the parameters listed in the Figure.

Infants enter the infant model (Supplemental Figure 2b) after birth, either as HIV-exposed but uninfected infants, or as infants infected during the intrauterine/intrapartum period. Exposed-uninfected infants face a monthly risk of breastfeeding transmission; this risk ceases at weaning or if maternal death occurs. From any infection state, infants face a risk of all-cause mortality. Monthly risks of infant HIV infection and infant mortality are stratified by the parameters listed in the Figure.

**Supplemental Figure 3. Maternal ART costs and proportion of women on ART following three key PMTCT strategies (assuming 100% uptake of services and 100% retention in care).**

**Supplemental Figure 3a.**

**Supplemental Figure 3b.**

**LEGEND**. Supplemental Figure 3, as well as manuscript Figures 1c and 1d, describe the impact of ART interruption on the number of postpartum HIV-infected women on ART after delivery, and thus on ART-related costs. Because clinic visits occur 3-monthly, the majority of costs are tallied in the model at 3-month intervals.

After the initial PMTCT regimens, HIV-related costs in the first five years are driven primarily by ART costs. Postnatal care strategies are similar following the “no antenatal ARVs,” sdNVP, and Option A strategies: women enrolled in HIV-related care are assumed to begin ART when CD4 falls below ≤350/µL or Stage 3-4 disease develops. Following Option B+, all women continue their three-drug regimens, while in Option B, women with CD4 >350/µL and no Stage 3-4 disease interrupt their ARVs, but remain in care and re-initiate ART once CD4 falls below ≤350/µL or Stage 3-4 disease develops.

Results from the CEPAC adult model show monthly ART costs for women not eligible for ART at delivery (CD4 >350/µL, no Stage 3-4 disease) under three postnatal care scenarios: 1) initiate three-drug ARVs in pregnancy and continue ARVs after weaning (as in Option B+); 2) initiate three-drug ARVs in pregnancy and interrupt ARVs after weaning (Option B); and 3) not initiate ARVs in pregnancy, but remain in care and initiate ART when needed (CD4 ≤350/µL or Stage 3-4 disease, as in the no antenatal ARVs, sdNVP, and Option A strategies). At 18 months after delivery, the proportion of women on three-drug ARVs in the Option B strategy falls to nearly 0 (Supplemental Figure 3a), as do the corresponding ART costs (Supplemental Figure 3b). By five years after delivery, ART costs in the Option B strategy are still much lower than in the Option B+ strategy, or following no antenatal ARVs, sdNVP, or Option A. Interrupting ART at weaning therefore saves money compared to continuing ART, however, this ART interruption may be associated with negative long-term health impacts for HIV-infected mothers.

**REFERENCES:**
